# Proteomics reveals dynamic metabolic changes in human hematopoietic stem progenitor cells from fetal to adulthood

**DOI:** 10.1186/s13287-024-03930-x

**Published:** 2024-09-15

**Authors:** Mingfang Xiong, Yanyu Xiu, Juan Long, Xiao Zhao, Qianqian Wang, Haoyu Yang, Hang Yu, Lihong Bian, Yan Ju, Hongyu Yin, Qingxiang Hou, Fei Liang, Nan Liu, Fudong Chen, Ruiwen Fan, Yuying Sun, Yang Zeng

**Affiliations:** 1 Senior Department of Hematology, the Fifth Medical Center of Chinese PLA General Hospital, Beijing, 100071 China; 2 Medical School of the Chinese PLA General Hospital, Beijing, 100039 China; 3 Department of Gynecology, the Fifth Medical Center of Chinese PLA General Hospital, Beijing, 100071 China; 4 Department of Obstetrics and Gynecology, PLA Rocket Force Characteristic Medical Center, Beijing, 100088 China; 5 School of Basic Medical Sciences and Forensic Medicine, Hangzhou Medical College, Hangzhou, 311399 China; 6 College of Veterinary Medicine, Shanxi Agricultural University, Taigu, 030801 Shanxi China

**Keywords:** Hematopoietic stem progenitor cells (HSPCs), Fetal liver (FL), Umbilical cord blood (UCB), Adult bone marrow (aBM), Proteomics, Metabolic remodelling, Glutathione (GSH)

## Abstract

**Background:**

Hematopoietic stem progenitor cells (HSPCs) undergo phenotypical and functional changes during their emergence and development. Although the molecular programs governing the development of human hematopoietic stem cells (HSCs) have been investigated broadly, the relationships between dynamic metabolic alterations and their functions remain poorly characterized.

**Methods:**

In this study, we comprehensively described the proteomics of HSPCs in the human fetal liver (FL), umbilical cord blood (UCB), and adult bone marrow (aBM). The metabolic state of human HSPCs was assessed via a Seahorse assay, RT‒PCR, and flow cytometry-based metabolic-related analysis. To investigate whether perturbing glutathione metabolism affects reactive oxygen species (ROS) production, the metabolic state, and the expansion of human HSPCs, HSPCs were treated with buthionine sulfoximine (BSO), an inhibitor of glutathione synthetase, and N-acetyl-L-cysteine (NAC).

**Results:**

We investigated the metabolomic landscape of human HSPCs from the fetal, perinatal, and adult developmental stages by in-depth quantitative proteomics and predicted a metabolic switch from the oxidative state to the glycolytic state during human HSPC development. Seahorse assays, mitochondrial activity, ROS level, glucose uptake, and protein synthesis rate analysis supported our findings. In addition, immune-related pathways and antigen presentation were upregulated in UCB or aBM HSPCs, indicating their functional maturation upon development. Glutathione-related metabolic perturbations resulted in distinct responses in human HSPCs and progenitors. Furthermore, the molecular and immunophenotypic differences between human HSPCs at different developmental stages were revealed at the protein level for the first time.

**Conclusion:**

The metabolic landscape of human HSPCs at three developmental stages (FL, UCB, and aBM), combined with proteomics and functional validations, substantially extends our understanding of HSC metabolic regulation. These findings provide valuable resources for understanding human HSC function and development during fetal and adult life.

**Supplementary Information:**

The online version contains supplementary material available at 10.1186/s13287-024-03930-x.

## Introduction

The self-renewal and differentiation properties of hematopoietic stem cells (HSCs) are responsible for the generation of mature blood lineages and the maintenance of the hematopoietic system throughout life. In mammals, the first HSCs emerge at the aorta-gonad-mesonephros (AGM) region, expand in the fetal liver (FL), and subsequently home to the fetal bone marrow (BM), which is the primary site of adult hematopoiesis [1, 2]. Both intrinsic and extrinsic mechanisms play critical roles in regulating HSC development. During hematopoietic development, hematopoietic cells undergo a series of phenotypical and functional changes [3, 4]. HSCs generate energy primarily via anaerobic metabolism and depend on a high level of glycolysis [5]; however, the overall metabolic properties of HSCs remain elusive, especially in humans. Major efforts have been dedicated to expanding HSPCs via ex vivo culture but have resulted in only modest expansion of long-term HSCs (LT-HSCs) [6]. Modifications of HSC metabolism during ex vivo culture may affect HSC quiescence and potency [7, 8].

Fetal and adult HSCs differ in their cell cycle status, molecular profile, metabolic state, and microenvironment depending on their developmental stage [1]. Compared with adult HSCs, murine FL HSCs display a rapid rate of expansion in vivo to expand the stem cell pool reasonably and supply differentiated blood cells within a short period during embryonic development [9, 10]. Compared with aBM HSCs, highly proliferative FL HSCs contain more mitochondria, causing elevated oxygen consumption levels and the production of ROS [11]. ROS have been implicated in HSC self-renewal potential and cell fate decisions [12, 13]. Maintaining low ROS levels in HSCs is pivotal for maintaining quiescence and preventing premature HSC differentiation [14]. Furthermore, the long-term reconstitution efficiency of HSCs in human neonate BM is greater than that of UCB [15]. These studies highlight the heterogeneity of HSCs during hematopoietic development. Recent findings revealed the independent origin and limited detectable expansion of murine FL HSPCs, which is challenging for the current dogma [16, 17].

Information on the different mechanisms of HSPC development has been gained mainly from studies in mouse models and in vitro systems, but they have yet to be validated in humans. It is generally impossible to isolate enough human immunophenotypic HSCs (Lin^−^CD34^+^CD38^−^CD90^+^CD45RA^−^CD49f^+^) and hematopoietic progenitor cells (HPCs) that can meet the current cell number requirements for proteome analysis due to the limited accessibility of human embryos and neonate blood samples. No more than a few thousand cells per specific phenotypic subpopulation could be FACS-sorted from a single sample in general [18]. Previous research has identified characteristic patterns of differentially expressed proteins (DEPs) in HSPCs derived from human granulocyte colony-stimulating factor (G-CSF)-primed peripheral blood mononuclear cells (PBMCs) [18, 19]. Proteomic data also revealed different characteristics of human BM CD34^+^ HSPCs from different ages [20], as well as the comparisons between HSPCs and leukemic stem cells (LSCs) [21, 22]. However, the proteomic characteristics of the fetal, newborn, and adult human HSPCs are still lacking, which is fundamental for understanding the development and regeneration of human HSCs.

As the molecular properties of human HSPCs have predominantly been studied at the transcript level [4, 15, 23, 24], knowledge about posttranscriptional mechanisms and associated networks remains elusive. Several posttranscriptional mechanisms have been implicated in regulating HSCs [25]; however, the transcriptome, proteome, and metabolome of the same cell population often vary significantly [21]. As the cellular functions and phenotypes of a cell are directly determined by its proteins, transcriptome characteristics may be insufficient to understand their mechanisms fully [26]. Thus, delineating the specific protein expression of human HSPCs is the key to identifying the functional regulators of human HSPCs during development.

Here, we established a metabolomic landscape of human lineage (Lin)^−^CD34^+^ HSPCs at both the FL, UCB, and aBM stages at the proteomic level, which could fill these critical gaps in our understanding of the metabolic regulation of human HSPCs. However, performing more in-depth comparisons of different subpopulations of human Lin^−^CD34^+^ HSPCs is currently difficult, as the cell numbers of subpopulations are scarce. Our findings on the proteome of human Lin^−^CD34^+^ HSPCs lay the foundation and provide indispensable clues for subsequent studies of immunophenotypic HSCs/HPCs after the optimization of proteome technologies. Our study also paves the way for understanding the development of HSCs during human hematopoiesis in vivo, providing valuable insights and strategies for generating clinically functional HSCs from iPSCs and their potential application for transplantation and therapy.

## Materials and methods

### Sample collection

Human embryonic samples from the initial trimester (9–10 post-conception weeks (PCW)) were acquired through elective medical pregnancy termination at the Fifth Medical Center of the PLA General Hospital within a 6-h window post-operation. These embryo samples were categorized by age following the standard crown–rump length (CRL) measurement. Anonymized heparinized samples of human UCB and aBM samples from haematological normal donors were obtained from the Fifth Medical Center of the PLA General Hospital and the Beijing Cord Blood Bank. The samples used for the experiments were FL (n = 22), UCB (n = 22), and aBM (n = 18). The summarized information of all of the samples collected in this study is shown in Additional file 2.

### Preparation of single-cell suspensions

The FL samples were collected within two hours after the termination of pregnancy, and the FL tissues were cut into small pieces and digested with collagenase VI for 15–20 min at 37 °C with periodic mixing. Then, the FL single-cell suspension was filtered through a 100 µm cell strainer. The FL, UCB, and aBM mononuclear cells (MNCs) were isolated by density gradient centrifugation according to the manufacturer’s protocol (Ficoll-Paque, GE Healthcare) and filtered through a 70 µm cell strainer. All experiments we performed were on freshly isolated samples within six hours to avoid the perturbations induced by the freezing and thawing of cells.

### Flow cytometry and MACS

After staining with CD34-PE (581, 343506, BioLegend) and CD45-FITC (HI30, 560976, BD), the proportion of CD34^+^CD45^mid^ cells was analyzed with a FACS Calibur. CD34^+^ cells were purified from FL, UCB, and aBM monocular layers via two rounds of magnetic positive cell selection (MACS, Miltenyi Biotec) with CD34 microbeads and MS columns (Miltenyi Biotec). Lin^−^CD34^+^ HSPCs were sorted on a Sony MA900 Multi-Application Cell Sorter. Human Lineage antibody cocktail including anti-human CD2, CD3, CD14, CD16, CD19, CD56, and CD235a (eFluor™ 450, clone: RPA-2.10, OKT3, 61D3, CB16, HIB19, TULY56, HIR2, 22-7775-72, eBiosciences). The FACS-sorted cells were washed with PBS and centrifuged to discard the supernatant, flash-frozen, and stored at − 80 °C for subsequent proteomics analysis.

To calculate the proportions of Ficoll-purified human FL, UCB, and aBM CD90^+^ HSCs (Lin^−^CD34^+^CD38^−^CD45RA^−^CD90^+^), we stained the MNCs with anti-human Lineage-eFluor™ 450, anti-human CD45-BV421 (HI30, 563879, BD), anti-human CD34-FITC (581, 343503, Biolegend), anti-human CD38-PE-Cy7 (HB-7, 356608, Biolegend), anti-human CD45RA-BV605 (5H9, 740424, BD), and anti-human CD90-APC (5E10, A15726, Life) antibodies. To compare the expression of surface markers of FL, UCB, and aBM HSPCs for FACS validation, we stained the MNCs with anti-human HLA-DR/DP/DQ-FITC (Tü39, 361705, BioLegend), anti-human CD133-APC (TMP4, 17-1338-42, eBioscience), and anti-human CD144-BV786 (55-7H1, 565672, BD). Flow cytometry was performed on a BD FACSymphony™ A5. Data analysis was performed using Flowjo V10.8.1 software (http://www.flowjo.com).

### Cell culture

Freshly isolated UCB-derived Lin^−^CD34^+^ cells were incubated with buthionine sulfoximine (BSO, 125 µM) or N-acetyl-L-cysteine (NAC, 100 µM) or both for 2 days in serum-free conditions with Stem-Span SFEM II (StemCell Technologies, 9655) supplemented with human TPO, SCF, and FLT3-L (50 ng/mL each) (Peprotech, AF-300-18, AF-300-07; AF-300-19) and 1% penicillin–streptomycin (P/S).

### Colony-forming assays

For methylcellulose colony-forming assays, 200 FACS-sorted HSPCs (Lin^−^CD34^+^CD38^–^) were plated with methylcellulose-based medium containing recombinant cytokines for human cells (Methocult, StemCell Technologies, H4434, contains SCF, IL-3, EPO, and GM-CSF) onto 24-well plates. Colonies were counted and morphologically assessed after 14 days.

### Cell cycle

Each group of cells labelled with human HSPC markers was washed twice with cold PBS. Cells were fixed and permeabilized with BD Cytofix/Cytoperm and stained with Ki67-PE (SolA15, 12-5698-82, eBioscience) and Hoechst33342 (10 µg/ml).

### Apoptosis analysis

Each group of cells labelled with human HSPC markers was washed twice with cold PBS and incubated with Annexin V (KeyGEN BioTECH) and 7-AAD (Invitrogen) for 30 min at 4 °C according to the manufacturer’s instructions. The proportion of apoptotic cells was detected by flow cytometry within 1 h.

### Seahorse assays

The oxygen consumption rate (OCR) and the proton efflux rate (PER) were measured using a Seahorse XFe96 Extracellular Flux Analyser (Agilent). OCR and PER were performed with a Seahorse XF Cell Mito Stress Test Kit (Agilent, 103015–100) and a Seahorse XF Glycolytic Rate Assay Kit (Agilent, 103344–100), respectively. A mixture of mitochondrial pressure medium was prepared by adding 200 µL of glucose (1 M), pyruvate (100 mM), and glutamine (200 mM) to 19.4 mL of RPMI medium (Agilent, 103576–100) and storing it according to the manufacturer's instructions. The glycolytic rate assay medium was similar to the medium above for measuring mitochondrial pressure. The OCR and PER were measured according to the manufacturer's protocol. Briefly, we pipetted 180 µL of a cell suspension of 1 × 10^5^ purified FL and UCB Lin^−^CD34^+^ HSPCs into each sample well of fibronectin (Gibco, 33016015)-coated 96-well XFe plates. After three baseline OCR measurements, the cells were sequentially exposed to oligomycin (1.5 µM), carbon-cyanide-4 (trifluoromethyl) phenazine (FCCP; 2 µM), and rotenone/antimycin A (Rot/AA; 1 µM). Three measurements were recorded after each injection. After three baseline PER measurements, the cells were sequentially exposed to a Rot/AA mixture (0.5 μM) and 2-deoxyglucose (2-DG; 50 mM). After normalization by cell number/well, the results were analyzed using the Wave program 2.6.3.5 (Agilent) and GraphPad Prism 9.

### Metabolic state analysis of HSPCs

Sorted FL, UCB, and aBM HSPCs (1 × 10^5^ per sample, n = 4) were lysed, and the intracellular GST activity was measured using a Glutathione S-transferase (GST) Activity Assay Kit (BC0355, Solarbio) according to the manufacturer's instructions. GSH/GSSG levels were assessed using a GSH and GSSG assay kit (S0053, Beyotime). For pyruvate content analysis, sorted FL and UCB HSPCs (1 × 10^5^ per sample, n = 3) were measured using a Pyruvate Assay Kit (BC2205, Solarbio) according to the manufacturer's instructions. For 2-NBDG (2-[N-(7-nitrobenz-2-oxa-1,3-diazol-4-yl)amino]-2-deoxy-D-glucose) glucose uptake, 5 × 10^6^ MNCs were first stained with human HSPC phenotypic markers (Lineage cocktail, CD34, CD38 antibodies) were stained with 50 μg/mL 2-NBDG (ab235976, Abcam) in 1 mL of PBS containing 0.5% BSA at 37 °C in incubation bins for 1 h, and flow cytometry was performed on a BD FACSymphony™ A5 (excitation/emission = 485/535 nm). For reactive oxygen species (ROS) analysis, 5 × 10^6^ MNCs were first stained with HSPC phenotypic markers and suspended with the CellROX^®^ Reagent (C10422, Invitrogen) at a final concentration of 5 μM and incubated for 30 min at 37 °C (excitation/emission = 640/665 nm).

### Measurement of the protein synthesis rate

A total of 5 × 10^6^ MNCs were first stained with HSPC phenotypic markers and mixed with 50 mM OP-Puro (C10456, Invitrogen) for 1 h, fixed for 20 min, and then detected with a Click-iT^Ⓡ^ OPP Reaction Buffer Kit (C10456, Invitrogen) according to the manufacturer’s instructions (excitation/emission = 495/519 nm).

### Mitochondrial analysis

The mitochondrial mass and active mitochondrial content were assessed by MitoTracker Green (MTG) (C1048, Beyotime) and MitoTracker Red CMXRos (C1035, Beyotime) staining, respectively. Briefly, 5 × 10^6^ MNCs were first stained with human HSPC phenotypic markers and then stained with MTG (100 nM) (excitation/emission = 490/516 nm) or MitoTracker Red CMXRos (200 nM) (excitation/emission = 579/599 nm) in 2 mL of HBSS (C1029, Beyotime) at 37 °C in incubation bins for 30 min.

### Sample preparation for proteomics analysis

Owing to the limited number of samples, the donor samples were analyzed in single replicates, and different individuals were considered biological replicates. The number of samples/donors was limited by the material available from the hospital for this study. We generated proteomic data for Lin^−^CD34^+^ HSPCs from FL (n = 4), UCB (n = 5), and aBM (n = 4) (10^5^ cells per sample) samples. All of the samples were processed via an in-solution digestion method with an EasyPept Ex very microprotein extraction kit (OSFP0004-8X, Omicsolution). In brief, a detergent solution (50 mM ammonium bicarbonate (ABC) solution containing 0.1% RapiGest and 0.4 μL of 5 × cocktail with a final concentration of 20 mM) was introduced to each sample and incubated for 2 h at 37 °C. Following cooling to room temperature, iodoacetamide (IAM) (40 mM) was added, and the mixture was mixed at 600 rpm for 1 min. This mixture was kept in darkness for 30 min. Subsequently, TEPC (Tris (2-carboxyethyl) phosphine) (3 µL) and CAA (chloroacetamide) (5 µL) were sequentially introduced into the mixture. The reaction was then terminated by heating to 95 °C, and trypsin (enzyme:protein = 1:2 (w/w)) digestion was carried out at 37 °C for 2 h. Finally, the peptides from each sample were purified, concentrated via vacuum centrifugation, and reconstituted in a solution containing 0.1% (v/v) formic acid. The peptide concentration was assessed by measuring the UV spectral density at 280 nm. For the data-independent acquisition (DIA) experiment, an index retention time (iRT) calibration peptide was included at a concentration of 400 ng per sample.

### MS assay for data-independent acquisition (DIA)

LC–MS/MS analysis was performed on a timsTOF Pro mass spectrometer (Bruker Daltonics, Bremen, Germany) coupled with a nanoElute liquid chromatograph (Bruker Daltonics, Bremen, Germany) with a 75 μm × 25 cm long column (Thermo Scientific) (1.9 μm id) for a 90 min gradient. Peptide separation was performed at a flow rate of 300 nL/min with mobile phases A (0.1% (v/v) formic acid in water) and B (0.1% (v/v) formic acid in acetonitrile) along with a 90 min gradient as follows: 0–70 min, 2–22% B; 70–78 min, 22–37% B; 78–83 min, 37–95% B; and 83–90 min, 95% B. The mass spectrometer was operated in positive ion mode and defined ion mobility MS from TIMS scans in a single 100 ms 100–1700.32 window mass range from the m/z-ion mobility plane. The collision energy increased linearly with mobility during parallel accumulation–serial fragmentation (PASEF) MS/MS scanning, ranging from 20 eV at 1/K0 = 0.60 Vs/cm^2^ to 59 eV at 1/K0 = 1.60 Vs/cm^2^.

### MS data analysis

The DIA data were analyzed using a database search of Spectronaut™ 14.4.200727.47784. The main software parameters were set as follows: the retention time prediction type was dynamic iRT, MS2 level correction interference was enabled, and cross-run normalization was enabled. All of the results were filtered according to the q value cut-off of 0.01 (equivalent to an FDR < 1%). The database information used in our search database was “Swissprot-Home-sapiens-20405-20230103".

### PCA

After normalization to the total peak intensity, the processed data were uploaded and imported into SIMCA-P (version 14.1, Umetrics, Umea, Sweden), where multivariate data analysis, including Pareto-scaled PCA, was performed.

### Pearson correlation coefficient

Pearson correlation coefficient analysis is a statistical method that measures the linear relationship between two variables by calculating the product-moment correlation coefficient between them. This correlation coefficient was between − 1 and + 1, where + 1 represented a complete positive correlation (one variable increased and the other increased).

### Cluster analysis

We conducted hierarchical clustering analysis employing cluster 3.0 (http://bonsai.hgc.jp/mdehoon/software/cluster/software.htm) and Java Treeview software (http://jtreeview.sourceforge.net). In hierarchical clustering, the Euclidean distance algorithm was used as the similarity measure, along with the average linkage clustering algorithm, which relies on the centroids of observed values. Additionally, visual representations in the form of heatmaps were provided alongside the dendrogram.

## Gene functional annotation analysis

### GO annotation

The protein sequences of the selected DEPs were locally searched for homologous sequences using the NCBI BLAST + client software (NCBI-BLAST-2.2.28 + -win32.exe), and internal scanning was performed to locate the GO terms and annotate the sequences. The GO annotation results were drawn using the R script.

### KEGG annotation

In accordance with the annotation procedure, the online KEGG database (http://geneontology.org/) was used to identify the KEGG homology of the studied proteins and localize them in the KEGG pathways.

### Enrichment analysis

Enrichment analysis was performed using Fisher’s exact test, which uses whole quantified proteins as the background dataset. Afterwards, Benjamini‒Hochberg correction was applied for multiple tests to adjust the derived *p* values. Only functional categories and pathways with a *p* value below the 0.05 threshold were considered significant.

### Fuzzy C-means clustering

The protein expression trends were illustrated to analyze the overall expression patterns of all DEPs at two consecutive stages among FL, UCB, and aBM HSPCs. Fuzzy processing was performed via Mfuzz software. The fuzzy c-means (FCM) algorithm was used for analysis, and all proteins were divided into nine different expression modules on the basis of their expression trends.

### Real-time quantitative (RT‒PCR)

We extracted total cellular RNA from Lin^−^CD34^+^ FL, UCB, and aBM cells using TRIzol reagent (Invitrogen, USA). The RNA was reverse transcribed to cDNA using the PrimeScript RT Reagent Kit (RR047A, Takara), subjected to PCR, and placed in a fluorescence quantitative PCR instrument for RT‒PCR data analysis.

*ENO1*-F: 5’-AGCCAGTGCAGGAATCCAGGTAG-3’

*ENO1*-R: 5’-ACTCGGTCACGGAGCCAATCT-3’

*PROM1*-F: 5’-CACTACCAAGGACAAGGCGTTCA-3’

*PROM1*-R: 5’-CGCTGGTCAGACTGCTGCTAAG-3’

*ESAM*-F: 5’-GGTGCTGTGCCTGTGATGGT-3’

*ESAM*-R: 5’-CCACTTCCTCTTCTCCTTCTGTCT-3’

*SDHB*-F: 5’-TCAGGAAGGCAAGCAGCAGTATCT-3’

*SDHB*-R: 5’-GATGGTGTGGCAGCGGTATAGAGA-3’

*GSTP1*-F: 5’-CCAGAACCAGGGAGGCAAGA-3’

*GSTP1*-R: 5’-GAGGCGCCCCACATATGCT-3’

*LANCL1*-F: 5’-TGAGTTCTCACAACGCTTGAC-3’

*LANCL1*-R: 5’-CGAGGGTCTGCTGATTTCAGG-3’

*GSTM2*-F: 5’-AAATGCTGAAGCTCTACTCACAGTTTC-3’

*GSTM2*-R: 5’-GGCTCAAATACTTGGTTTCTCTCAAG-3’

*GSS*-F: 5’-GGAACATCCATGTGATCCGAC-3’

*GSS*-R: 5’-GCCATCCCGGAAGTAAACCA-3’

*β-actin*-F: 5’-TCCATCATGAAGTGTGACGT-3’

*β-actin*-R: 5’-GAGCAATGATCTTGATCTTCAT-3’.

### Statistical analysis

All experiments were repeated at least three times independently; for all experiments, comparisons between two groups were performed via standard two-tailed Student’s *t* tests. We performed ordinary one-way ANOVA with Tukey's multiple comparison test to analyze the statistical significance of differences between more than two groups. A value of *p* < 0.05 was considered significant. Data with statistical significance (*p < 0.05, **p < 0.01, ***p < 0.001, and ****p < 0.0001) are shown in the figures.

## Results

### Proteomic profiling of human HSPCs from fetal to adult stages

**Fig. 1:**
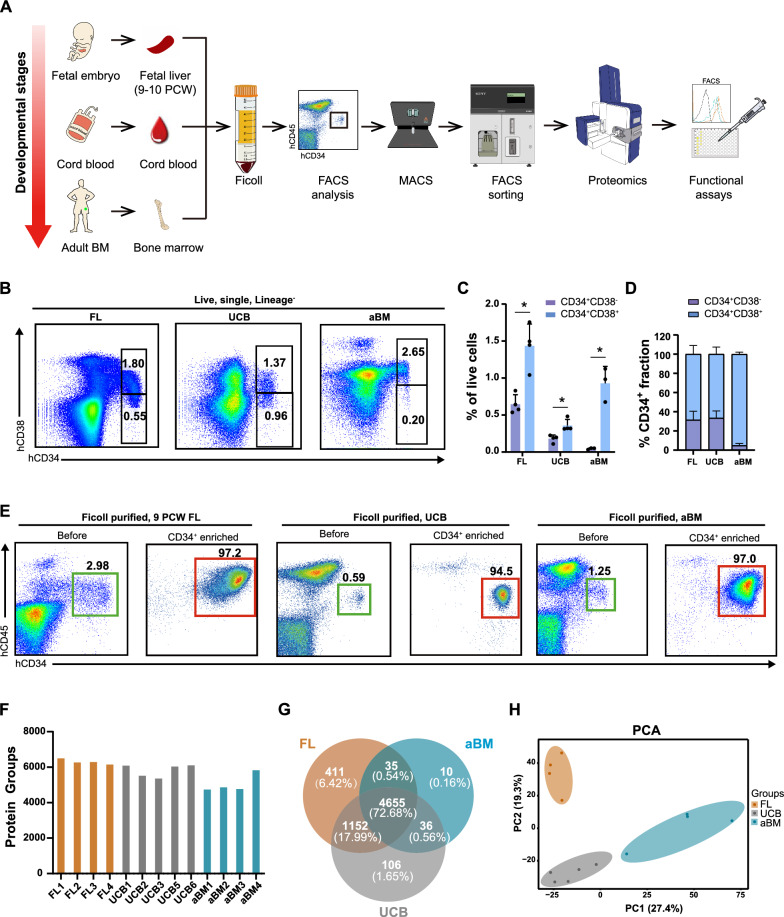
Proteomic profiling of human HSPCs at different developmental stages. **A** Schematic diagram of the experimental design. Lin^−^CD34^+^ HSPCs were derived from human FL, UCB, and aBM stages. The original proportion of Lin^−^CD34^+^CD45^mid^ HSPCs was analyzed via FACS. The MACS enrichment efficiency was confirmed by FACS again after sorting. The proteome of human Lin^−^CD34^+^ HSPCs was subsequently analyzed. Finally, relevant functional verification, including Seahorse, RT‒PCR, metabolic-related assays, and cell culture assays, were conducted. **B** Representative flow cytometry profiles of FL, UCB, and aBM cells plotted as hCD34 and hCD38 from live, single, lineage negative cells. **C** Percentages of Lin^−^CD34^+^CD38^−^/Lin^−^CD34^+^CD38^+^ cells among live FL, UCB, and aBM cells (FL, n = 4; UCB, n = 4; aBM, n = 3). The error bars represent the mean ± SD; the significance is indicated by paired Student’s *t* tests. *p < 0.05. **D** Percentages of the CD38^−^ and CD38^+^ fractions in FL, UCB, and aBM Lin^−^CD34^+^ cells (FL, n = 4; UCB, n = 4; aBM, n = 3). The error bars represent the mean ± SD. **E** Flow cytometry analysis before and after CD34 enrichment in FL, UCB, and aBM CD34^+^CD45^mid^ HSPCs. **F** Overview of the protein numbers of FL, UCB, and aBM HSPCs identified via DIA. **G** Venn diagram of proteins identified in FL (brown), UCB (dark grey), and aBM (peacock blue) samples. **H** PCA for the indicated comparison using all quantified proteins of FL (brown), UCB (dark grey), and aBM (peacock blue). PCA1 and PCA2 denote the first and second principal components, respectively.

We first investigated the differences in the protein characteristics of human HSPCs by generating single-cell suspensions from human FLs of 9–10 PCW, UCB, and aBM samples (Fig. 1A). MNCs from FL, UCB, and aBM were enriched by Ficoll density gradient separation. CD34 is a well-known common marker of human HSPCs, as validated by human clinical transplantation and xenograft transplantation assays [27]. CD34^+^ HSPCs are still a mixture of heterogeneous populations that contain CD34^+^CD38^−^ noncommitted progenitors (HSCs and multipotent progenitors [MPPs]) and CD34^+^CD38^+^ lineage-committed progenitors. Moreover, only a small minority of CD34^+^ cells are true HSCs [28]. We subsequently analyzed the composition of HSPCs and HSC frequency at different developmental stages (Fig. 1B, Additional file 1: Fig. S1A-C). The percentages of lin^−^CD34^+^CD38^−^ cells in FL, UCB, and aBM among total live cells were 0.65 ± 0.13%, 0.18 ± 0.05%, and 0.05 ± 0.01%, respectively, whereas the percentages of lin^−^CD34^+^CD38^+^ cells were 1.43 ± 0.30%, 0.36 ± 0.08%, and 0.93 ± 0.23%, respectively (Fig. 1C/1D). The proportions of human FL, UCB, and aBM HSCs (Lin^−^CD34^+^CD38^−^CD90^+^CD45RA^−^) [29, 30] were 0.14 ± 0.04%, 0.02 ± 0.002%, and 0.01 ± 0.006%, respectively (Additional file 1: Fig. S1D), which are capable of long-term multilineage transplantation according to previous studies.

To obtain enough CD34^+^ HSPCs with high purity, the MNCs from FL, UCB, and aBM were magnetically enriched for CD34^+^ cells using the human CD34 microbeads kit [24]. Then, Lin^−^CD34^+^ HSPCs were sorted after they were Ficoll-purified and CD34-enriched (Fig. 1E). The expression of the CD45 antigen in lineage blood cells is higher than that in HSCs/HPCs [24]. We also confirmed that Lin^−^CD34^+^ HSPCs derived from Ficoll-purified CD34^+^ MNCs were restricted to HSPCs and excluded from CD34^+^CD45^−^ endothelial cells (ECs) and lineage-positive (CD2, CD3, CD14, CD16, CD19, CD56, and CD235a) cells [31, 32]. Giemsa staining also confirmed that the CD34^+^CD14^−^ population presented with typical blast/stem cell characteristics of a relatively high nucleus-to-cytoplasm ratio [31]. We generated the proteome data from FL (n = 4), UCB (n = 5), and aBM (n = 4) samples (10^5^ Lin^−^CD34^+^ cells per sample).

Between 4,739 and 6,499 proteins (an average of 5,730 proteins per cell) were robustly quantified for each sample by data-independent acquisition (DIA) (Fig. 1F). Compared with previously published studies on human aBM HSPCs, the protein numbers identified in our datasets were comparable [18, 21]. The number of overlapping proteins identified among the three cell populations was 4,655 (72.68%) (Fig. 1G, Additional file 5). In addition, 411 proteins (6.42%) were exclusively detected in FL HSPCs (defined as “fetal-specific”), 106 proteins (1.65%) were identified as “newborn-specific”, and only 10 proteins (0.16%) were “adult-specific”. As expected, principal component analysis (PCA) revealed clear segregation between HSPCs at distinct developmental stages (Fig. 1H). Pearson correlation coefficient analysis between biological replicates in each of the three stages yielded, on average, *r* = 0.863 (± 0.058), indicating consistent measurement and high reproducibility of the whole workflow (Additional file 1: Fig. S2A).

### Subsets of human HSPC proteins undergo fetal-to-adult transition with different patterns

**Fig. 2:**
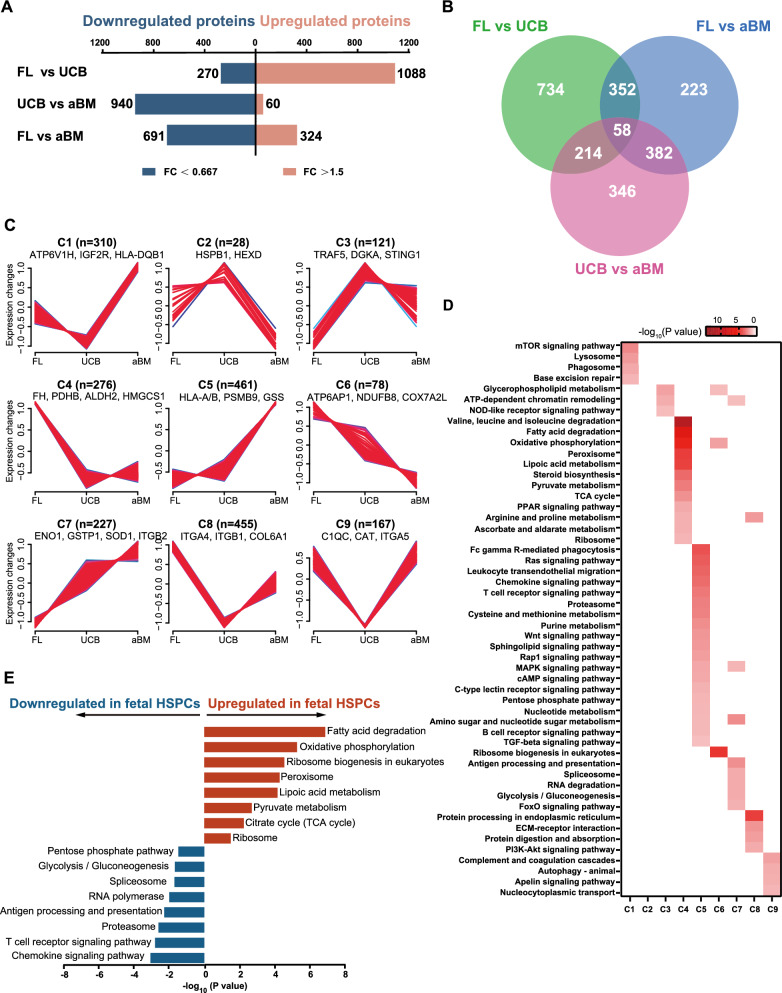
Differential protein levels throughout human HSPC development. **A** Changes in the proteomics of upregulated and downregulated proteins between each pair in FL, UCB, and aBM HSPCs. DEPs were analyzed at thresholds of FC > 1.5 or FC < 0.67 and *p* < 0.05. **B** Overlapping DEPs between each pair of stages: FL vs. UCB, UCB vs. aBM, and FL vs. aBM. **C** Mfuzz analysis is divided into nine different expression trends on the basis of the expression trends of all DEPs of each stage for human HSPCs. The X-axis represents the other groups, and the Y-axis represents the expression change after homogenization. The lines of each cluster refer to a class of proteins expressed in proteins. Each cluster identifies its representative proteins. **D** Heatmap showing the -log_10_ transformed significant value (*p* value) of the KEGG pathway terms describing each of the 9 clusters. **E** KEGG pathway enrichment analysis of terms significantly upregulated and downregulated in fetal HSPCs versus UCB and aBM HSPCs

Next, we calculated the DEPs between HSPCs at different developmental stages to identify their protein characteristics (fold change > 1.5 or < 0.667 and *p* < 0.05) (Fig. 2A, Additional file 1: Fig. S2B, Additional file 3). The subcellular distribution of DEPs was shown in Additional file 1: Fig. S2C. These DEPs were used for downstream analyses. Statistical analysis revealed 1,358 DEPs between fetal and newborn HSPCs. The most significant fraction (1,088 proteins) of these proteins was highly expressed in FL HSPCs. Surprisingly, compared with FL or aBM HSPCs, UCB HSPCs presented very few DEPs, which suggested that UCB HSPCs presented more similar protein expression features to FL and BM HSPCs but fewer unique expression features. The proteins differentially expressed between different pairs of cell populations were analyzed via a Venn diagram (Fig. 2B, Additional file 1: Fig. S4A–B, Additional file 5). To identify the dynamic changes in HSPC development between neighbouring cell populations, fuzzy c-means (FCM) [33] was applied to cluster global protein expression profiles. We observed nine distinct clusters of protein expression patterns representing proteins that were differentially regulated (Fig. 2C, Additional file 1: Fig. S3, Additional file 4). Among these, clusters 4 and 6 represented proteins upregulated in fetal HSPCs and then downregulated after birth. In contrast, clusters 5 and 7 represented proteins that were downregulated in fetal HSPCs and upregulated after birth.

Kyoto Encyclopedia of Genes and Genomes (KEGG) pathway analysis was subsequently applied to identify pathways enriched in each cluster. Notably, fatty acid degradation, oxidative phosphorylation (OXPHOS), the tricarboxylic acid (TCA) cycle, the peroxisome proliferator-activated receptor (PPAR) signaling pathway, and ribosome were enriched in clusters 4 or 6, with higher expression in fetal HSPCs (Fig. 2D–E). In contrast, chemokines, immune-related pathways (such as T and B-cell receptors), signalling pathways (Wnt, MAKP, TGF-β, and FoxO), antigen processing and presentation, proteasome, spliceosome (involved in mRNA maturation), glycolysis/gluconeogenesis, and the pentose phosphate pathway (PPP) were among the top differentially regulated pathways enriched in clusters 5 or 7, showing an upregulated pattern during human HSPC development (Fig. 2D–E). The FoxO signalling pathway, which plays essential roles in response to physiological oxidative stress and the detoxication of ROS [34], was active in UCB and aBM HSPCs but not in FL HSPCs (Fig. 2D).

Protein translational machinery-related pathways, such as ribosome and ribosome biogenesis, were more enriched in FL HSPCs than in UCB and aBM HSPCs (Fig. 2D–E, Additional file 1: Fig. S3A). Both ribosomal assembly and protein synthesis have been implicated in HSC function and regeneration [26, 35]. A low level of protein synthesis is essential for maintaining HSC metabolic homeostasis, whereas an alteration in protein synthesis impairs HSC function [26, 36]. Homeostatic or quiescent HSCs restrict protein synthesis for long-term maintenance. In contrast, proliferating HSCs undergo excessive protein synthesis to support their drastic expansion, which may increase the number of misfolded proteins and impair HSC function. Human FL hematopoiesis requires a highly regulated protein synthesis rate; it acts as an essential niche for hematopoietic differentiation and HSC expansion until birth [1, 32]. In addition, peroxisome-related proteins were also upregulated in FL HSPCs, whereas the spliceosome and proteasome were highly expressed in UCB and aBM HSPCs (Fig. 2D–E, Additional file 1: Fig. S3A).

Taken together, our proteome data revealed dynamic and complex protein expression patterns during the fetal-to-adult transition process in human HSPC development, which occurred progressively along with a continuum of HSPC maturation.

### Metabolic switch of human HSPCs from fetal to adulthood through the perinatal period

**Fig. 3:**
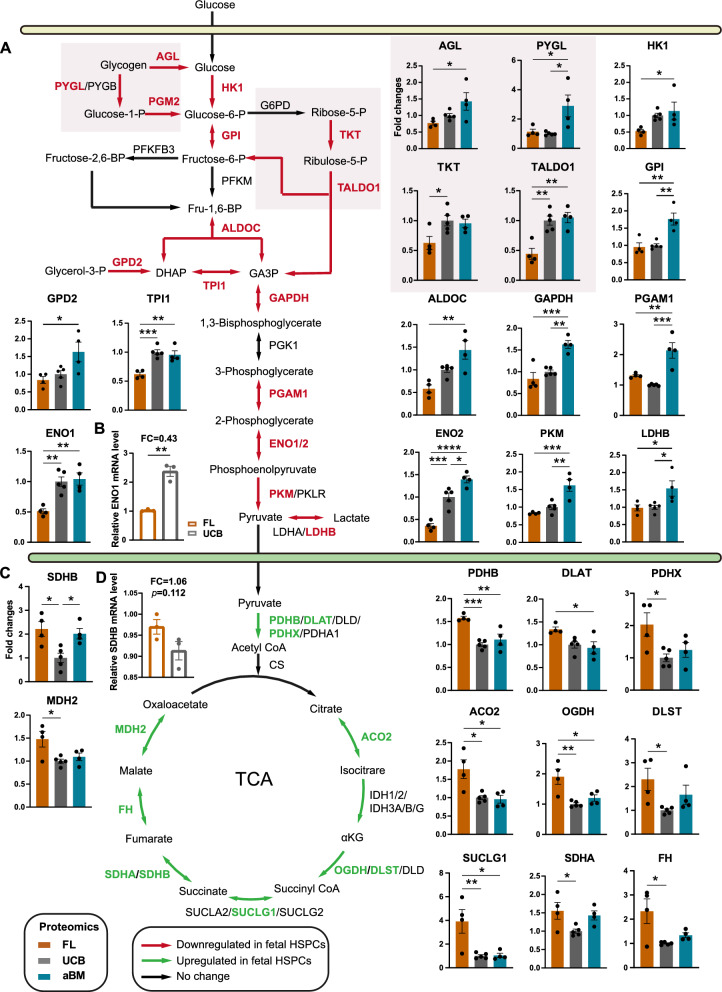
Prominent changes in central carbon metabolism occur in HSPCs at three developmental stages. **A**–**B** Glucose metabolism (glycogen metabolism and PPP) **A** and the TCA cycle **B** in FL (brown), UCB (dark grey), and aBM (peacock blue) HSPCs are depicted as unidirectional arrows representing unidirectional reactions and bidirectional arrows indicating bidirectional reactions. The gene names of the respective enzymes are written in capital letters. Green indicates the enzymes upregulated in fetal HSPCs, red indicates the enzymes downregulated in fetal HSPCs, and black indicates which enzyme was not changed (see also Fig. 4). Fold change (FC) proteomics expression data of enzymes are shown as histograms in the indicated comparisons. Four to six independent experiments were performed per enzyme. The significance of the proteomic data is indicated by one-way ANOVA with Tukey’s multiple comparison test. *p < 0.05, **p < 0.01, ***p < 0.001, and ****p < 0.0001. **C**–**D** RT‒PCR analysis (n = 3) of ENO1 and SDHB in FL and UCB HSPCs. The significance is indicated by an unpaired Student’s *t* test. *p < 0.05 and **p < 0.01

Compared with HSPCs in the UCB or aBM stages, those in the FL stage proliferate extensively, resulting in different metabolic demands [24]. However, the metabolic pathways responsible for the production of energy and protein synthesis at the human fetal, newborn, and adult stages have not been described comprehensively. As shown in Fig. 2E and Additional file 1: Fig. S3B, the most notable proteome differences between HSPCs at the three different developmental stages were related to central carbon metabolism (summarized in Fig. 3). Proteome analysis revealed that the rate-limiting enzymes involved in facilitating the entry of glucose into glycolysis/gluconeogenesis, such as hexokinase 1 (HK1), glycogen phosphorylases liver form (PYGL), phosphoglucose mutase 2 (PGM2), glycolytic enzymes aldolase C (ALDOC), triosephosphate isomerase (TPI1), and enolase-1/2 (ENO1/2), were overexpressed in UCB or aBM HSPCs (Fig. 3A). These findings indicated that adult HSPCs rely primarily on glycolysis to supply energy. HK1, the enzyme that catalyses the first rate-limiting step of glycolysis, exhibited an age-associated increase among human BM CD34^+^ HSPCs [20]. In addition, we detected a significantly increased level of ENO1 in UCB HSPCs compared with their FL counterparts via RT‒PCR (Fig. 3B). The quiescent HSPCs have low energy requirements and are believed to depend on glycolysis rather than mitochondria. They are sustained by a hypoxic ecological niche and display great self-renewal capacity [37–39].

**Fig. 4:**
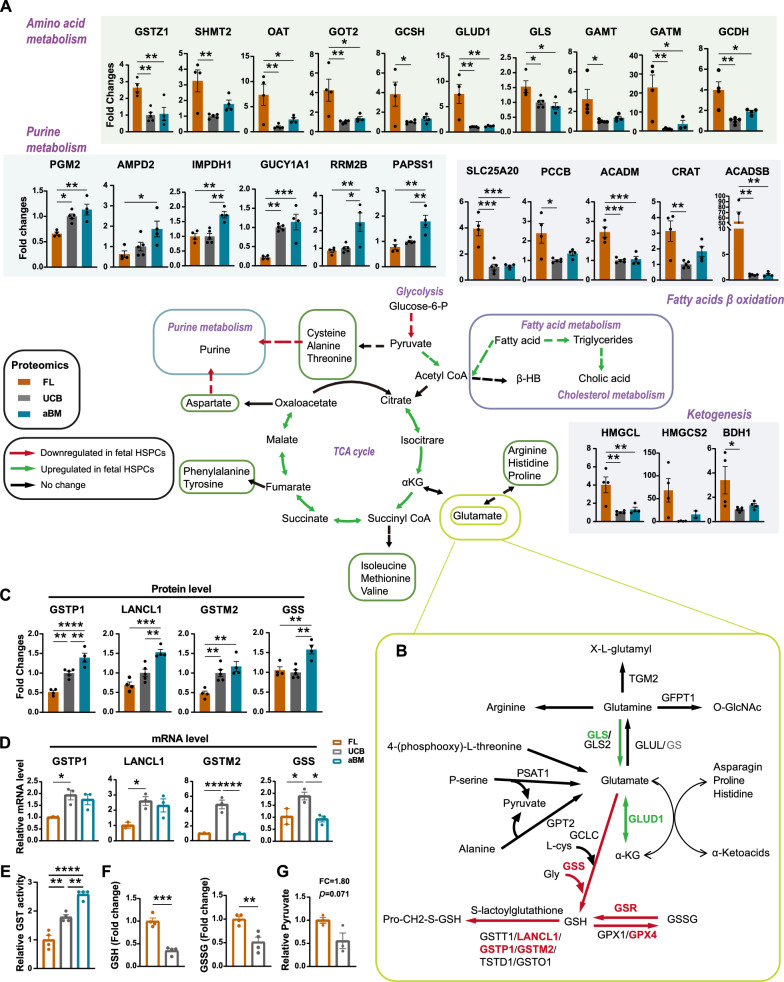
The metabolic landscape of human HSPCs among FL, UCB, and aBM. **A** Changes in purine, AA, fatty acid, ketogenesis, glutamine, and glutathione metabolism between FL (brown), UCB (dark grey), and aBM (peacock blue) HSPCs. Green indicates the enzymes upregulated in fetal HSPCs (see Fig. 3). Fold changes in the proteomeo data of enzymes are shown as histograms in the indicated comparison. Four to six independent experiments were performed per enzyme. The significance of the proteomic data is indicated by one-way ANOVA with Tukey’s multiple comparison test. *p < 0.05, **p < 0.01, and ***p < 0.001. **B** Scheme of glutamine-related metabolism and glutathione metabolism replenishment. **C** Proteomic expression data of glutathione metabolism-related enzymes are shown as histograms. The significance of the proteomic data is indicated by one-way ANOVA with Tukey’s multiple comparison test. *p < 0.05, **p < 0.01, ***p < 0.001, and ****p < 0.0001. **D** RT‒PCR analysis (n = 3) of GSTP1, LANCL1, GSTM2, and GSS in FL and UCB HSPCs. *p < 0.05, **p < 0.01, and ***p < 0.001. **E** GST levels of FL, UCB, and aBM HSPCs. Significance is indicated by one-way ANOVA with Tukey’s multiple comparison test. *p < 0.05, **p < 0.01, ***p < 0.001, and ****p < 0.0001. **F** GSH and GSSG levels in FL and UCB HSPCs. Significance is indicated by the Student’s *t* test. *p < 0.05, **p < 0.01, and ***p < 0.001. **G** Pyruvate levels in FL and UCB HSPCs as indicated (n = 3)

In contrast, the proteins involved in OXPHOS and the TCA cycle presented increased expression in FL HSPCs (Fig. 3C). Pyruvate dehydrogenases (PDHB and PDHX) were highly expressed in FL HSPCs, promoting entry into the TCA cycle [40]. Furthermore, FL HSPCs presented increased pyruvate levels, although the difference was insignificant (Fig. 4G), consistent with increased mitochondrial OXPHOS. The enzymes involved in the TCA cycle (such as ACO2, IDH1, SDHA, SDHB, and FH) were abundant at the fetal stage (Fig. 3C-D). FH has been demonstrated to be a critical metabolic regulator of HSC self-renewal and differentiation [41]. These significantly differentially expressed enzymes could be targeted to intervene in the energy metabolism of human HSPCs and further influence their fate.

In addition to these mitochondria-related pathways, amino acid (AA) metabolism (especially valine, arginine, and proline), fatty acid degradation, lipoic acid metabolism, and steroid biosynthesis were among the pathways most differentially regulated in FL HSPCs compared with those in UCB or aBM HSPCs (Fig. 2D/4A). Enzymes involved in glutamine metabolism, such as glutaminase (GLS) and glutamate dehydrogenase 1 (GLUD1), were highly enriched in FL HSPCs (Fig. 4B). However, the enzymes that participate in glutathione (GSH) metabolism, such as S-transferases (GSTs), GSTP1, LANCL1, and GSTM2, as well as the GSH synthesis enzymes GSR and GPX4, presented increased expression patterns in UCB or aBM HSPCs (Fig. 4B-C). We also detected significantly increased levels of GSTP1, LANCL1, GSTM2, and GSS in UCB or aBM HSPCs compared with their FL counterparts via RT‒PCR (Fig. 4D). Differences in mRNA expression were detected mainly before and after birth, not between newborn and adult HSPCs. In addition, UCB and aBM HSPCs displayed high GST activity (Fig. 4E). Interestingly, compared with UCB HSPCs, FL HSPCs presented significantly increased levels of free glutathione pools, both reduced (GSH) and oxidized (GSSG) (Fig. 4F). We hypothesize that UCB HSPCs may have a higher glutathione transfer metabolism rate to consume GSH and GSSG than FL HSPCs, which needs further investigation.

### Switching from the oxidative pathway to the glycolytic pathway coincides with the development of human HSPCs

**Fig. 5:**
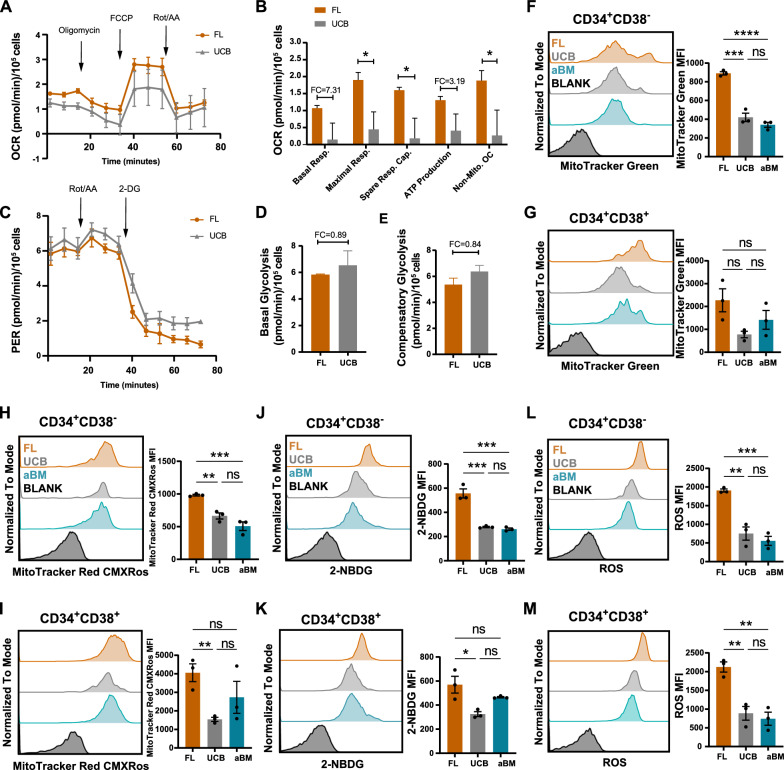
Higher mitochondrial function and increased glucose uptake support the oxidative consumption of FL HSPCs. **A** OCRs were examined in FL and UCB HSPCs via the Seahorse assay. The error bars represent the mean ± SEM. **B** Levels of basal respiration, maximal respiration, spare respiratory capacity, ATP production, and nonmitochondrial oxygen consumption were examined. The error bars represent the mean ± SEM; the significance is indicated by unpaired Student’s *t* tests. *p < 0.05. **C** PERs of FL and UCB HSPCs were measured. The error bars represent the mean ± SEM. **D**–**E** The levels of basal glycolysis (**D**) and compensatory glycolysis (**E**) were examined. The error bars represent the mean ± SEM. **F**–**G** Flow cytometric analysis of 2-NBDG glucose uptake in Lin^−^CD34^+^CD38^−^ (**F**) and Lin^−^CD34^+^CD38^+^ (**G**) cells at three developmental stages. **H**–**I** Flow cytometric analysis of total mitochondrial mass (MTG) in Lin^−^CD34^+^CD38^−^ (**H**) and Lin^−^CD34^+^CD38^+^ (**I**) cells at three developmental stages. **J**–**K** Flow cytometric analysis of the active mitochondrial content (MitoTracker Red CMXRos) in Lin^−^CD34^+^CD38^+^ (**J**) and Lin^−^CD34^+^CD38^−^ (**K**) cells at three different developmental stages. **L**–**M** Flow cytometric analysis of cellular ROS levels in Lin^−^CD34^+^CD38^−^ (**L**) and Lin^−^CD34^+^CD38.^+^ (**M**) cells at three developmental stages. Significance is indicated by one-way ANOVA with Tukey’s multiple comparison test. *p < 0.05, **p < 0.01, ***p < 0.001, and ****p < 0.0001

Seahorse analysis was used to measure the oxygen consumption rate (OCR), which was indicative of OXPHOS. In contrast, the proton efflux rate (PER) in the glycolytic rate assay provides accurate measurements of glycolytic rates under basal conditions and compensatory glycolysis following mitochondrial inhibition, which indicates glycolysis. As shown in Fig. 5A-E, compared with FL HSPCs, UCB HSPCs demonstrated a lower OCR rate, basal respiration, maximal respiration, spare respiratory capacity, ATP production, and nonmitochondrial oxygen consumption. In contrast, UCB HSPCs displayed relatively greater basal glycolysis and compensatory glycolysis via PER measurements. Taken together, these findings are consistent with our findings that FL HSPCs primarily use OXPHOS to meet their energy demands and have a more active metabolic state than UCB HSPCs [11].

As the proteomic profile and Seahorse analysis revealed an increased mitochondrial respiratory rate in fetal HSPCs, we investigated whether human HSPCs at different developmental stages differed in terms of mitochondrial content and activity, glucose uptake, ROS production, and ribosomal translation. To obtain a sufficient number of cells for assessing mitochondrial function, we focused on the Lin^−^CD34^+^CD38^−^ population. MTG and MitoTracker Red CMXRos were used to quantify the total and active mitochondria, respectively (Fig. 5F/5H, Additional file 1: Fig. S5A-B). We also measured glucose uptake using 2-NBDG, a fluorescently tagged glucose analogue (Fig. 5J). Protein synthesis was analyzed via an O-propargyl-puromycin (OP-puro) incorporation assay (Additional file 1: Fig. S5G-H). FL Lin^−^CD34^+^CD38^−^ showed higher total and active mitochondria content than that in its UCB and aBM counterparts. An apparent increase in ROS production was noted in the FL Lin^−^CD34^+^CD38^−^ population compared with that in the UCB and aBM populations (Fig. 5L). The level of glucose uptake also increased in the FL Lin^−^CD34^+^CD38^−^ population. The same pattern was also shown in the Lin^−^CD34^+^CD38^+^ HPC population (Fig. 5G/5I/5K/5M), which was consistent with our proteomic analysis of the Lin^−^CD34^+^ HSPC population (Additional file 1: Fig. S5C-F).

### ***Distinct response of human Lin***^***−***^***CD34***^+^***CD38***^***−***^*** HSPCs and Lin***^***−***^***CD34***^+^***CD38***^+^***HPCs to perturb glutathione metabolism***

**Fig. 6:**
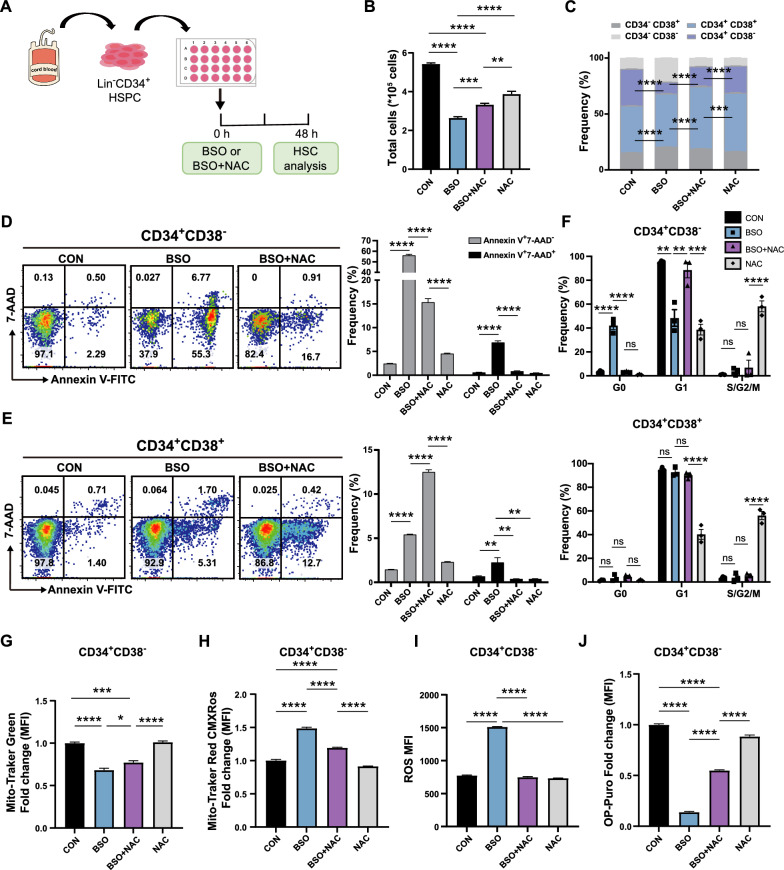
Distinct response of human HSCs and HPCs to disturb glutathione metabolism. **A** Experimental diagram of BSO/NAC/BSO + NAC treatment of human CD34^+^ HSPCs in vitro. **B** Number of total CD34^+^ HSPCs after culture in vitro for 48 h with BSO/NAC/BSO + NAC. **C** Frequency of CD34^+^CD38^−^ and CD34^+^CD38^+^ cells among CD34^+^ HSPCs after 48 h of culture with BSO/NAC/BSO + NAC. **D**–**E** Apoptosis analysis of HSPCs cultured for 48 h with BSO/NAC/BSO + NAC. Early (Annexin V^+^7-AAD^−^) and late (Annexin V^+^7-AAD^+^) apoptosis were quantified by flow cytometry in Lin^−^CD34^+^CD38^−^ HSPCs (**D**) and Lin^−^CD34^+^CD38^+^ HPCs (**E**). **F** Cell cycle analysis of CD34^+^CD38^−^ (up) and CD34^+^CD38^+^ (down) cells among CD34^+^ HSPCs after 48 h of culture with BSO/NAC/BSO + NAC. **G**–**J** Flow cytometric analysis of total mitochondria (MTG) (**G**), active mitochondrial content (MitoTracker Red CMXRos) (**H**), ROS levels (**I**), and protein synthesis rate (OP-Puro) (**J**) in Lin^−^CD34^+^CD38^−^ HSCs. (Error bars represent the means ± SEM, n > 3). Significance was indicated by one-way ANOVA with Tukey’s multiple comparison test (n > 3, *p < 0.05, **p < 0.01, ***p < 0.001, and ****p < 0.0001)

The enzymes (GSTP1, GSTM2, and LANCL1) involved in glutathione metabolism showed increased expression patterns in human HSPCs after birth (Fig. 4C-D). We investigated whether perturbing glutathione metabolism affects ROS production, the metabolic state, and the expansion of human HSPCs (Fig. 6A). BSO, an inhibitor of glutathione synthetase, can lead to an increase in intracellular ROS levels [42]. Treatment with BSO (125 µM) in vitro for two days significantly decreased HSC expansion (Lin^−^CD34^+^CD38^−^), leading to differentiation and significantly increased levels of ROS (Fig. 6B/6C/6I). Next, we asked whether the pharmacological inhibition of ROS elevation could protect human HSPCs from functional degradation. The antioxidant agent N-acetyl-L-cysteine (NAC, 100 µM) protected human HSPCs from BSO-induced HSPC differentiation and elevated ROS levels (Fig. 6B/6C/6I).

Unexpectedly, the apoptosis of HSPCs (Lin^−^CD34^+^CD38^−^) and progenitors (Lin^−^CD34^+^CD38^+^) in response to BSO and NAC was significantly different (Fig. 6D-E). The increase in BSO-induced elevated ROS levels significantly increased the number of apoptotic Lin^−^CD34^+^CD38^−^ HSPCs (FC = 20.85, *p* < 0.0001, relative to CON), and NAC rescued the cells from apoptosis (FC = 5.36, *p* < 0.0001, relative to CON) (Additional file 1: Fig. S6A). This effect appears to be specific to primitive Lin^−^CD34^+^CD38^−^ cells, as no drastic increase in apoptosis was observed in Lin^−^CD34^+^CD38^+^ HPCs (FC = 3.60, *p* < 0.0001, relative to CON). In contrast, NAC aggravated the apoptosis of HPCs (FC = 6.05, *p* < 0.0001, relative to CON) (Additional file 1: Fig. S6A). Interestingly, BSO treatment decreased the total mitochondrial content while increasing the active mitochondrial content in both the HSPC and HPC populations (Fig. 6G-H, Additional file 1: Fig. S6B-C). These results were also validated by quantifying the cellular ROS levels (Fig. 6I, Additional file 1: Fig. S6D-E). BSO treatment halted the cell cycle in Lin^−^CD34^+^CD38^−^ cells but not in Lin^−^CD34^+^CD38^+^ cells (Fig. 6F). BSO treatment of human CD34^+^ cells also significantly decreased de novo translation (protein synthesis rate), which was confirmed using OP-Puro (Fig. 6J, Additional file 1: Fig. S6F-G).

Taken together, these results demonstrated that the oxidative stress induced by BOS treatment led to ROS accumulation, a decreased protein synthesis rate, and the collective induction of apoptosis in Lin^−^CD34^+^CD38^−^ cells. In contrast, Lin^−^CD34^+^CD38^+^ cells could resist apoptosis to some extent. The antioxidant NAC also induced different responses to rescue BSO-induced elevated apoptosis in HSPCs and HPCs. Glutathione metabolism and ROS play critical roles in human HSC function (especially in mitochondria), and the regulation of glutathione-related metabolism and ROS has the potential to maintain HSC function and longevity and improve HSC regeneration or HSCT in patients.

### Progressive HSPC maturation progresses from the FL stage to the adult stage

**Fig. 7:**
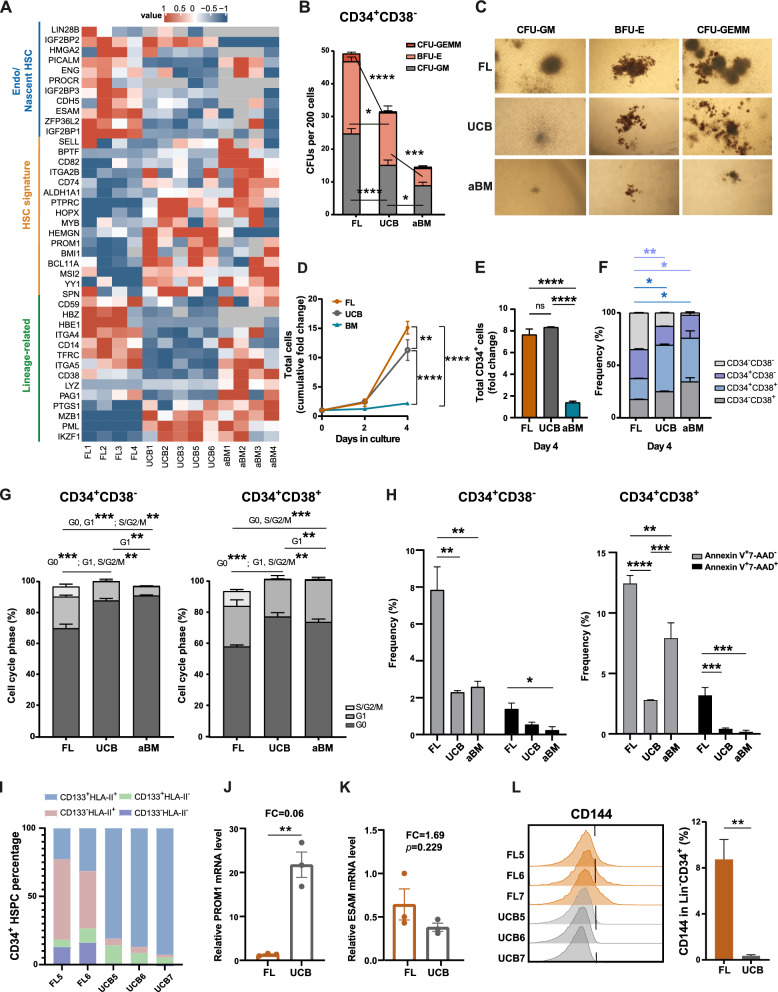
Functional differences between HSPCs from FL, UCB, and aBM. **A** Heatmap showing the scaled expression of endo/nascent HSCs, the HSC signature, and lineage-related signature proteins in FL, UCB, and aBM HSPCs. **B** Colony-forming cells (CFUs) per 200 cells from Lin^−^CD34^+^CD38^−^ HSPCs derive from three developmental stages after 14 days of culture (n = 3 biological independent samples in each group, n = 3–5 technical replicates of each sample). **C** Representative images of CFU-GM, BFU-E, and CFU-GEMM scored under an inverted microscope are shown (magnification × 40). Colony-forming units granulocyte–macrophage (CFU-GM), Burst forming units erythroid (BFU-E), Colony-forming units granulocyte/erythrocyte/macrophage/megakaryocyte (CFU-GEMM). **D** Cumulative in vitro total nucleated cell fold change of cultured Lin^−^CD34^+^ cells. **E** Relative CD34^+^ cell fold change after 4 days ex vivo. **F** Frequencies of CD34^+^CD38^−^ and CD34^+^CD38^+^ cells after 4 days of ex vivo expansion. **G** Percentage (mean ± SEM) of Lin^−^CD34^+^CD38^−^ (left) and Lin^−^CD34^+^CD38^+^ cells (right) in the indicated cell cycle phases as determined by flow cytometry (G0, Ki67^−^Hoechst33342^−^; G1, Ki67^+^Hoechst33342^−^; S/G2/M, Ki67^+^Hoechst33342^+^). **H** Apoptosis analysis of Lin^−^CD34^+^CD38^−^ (left) and Lin^−^CD34^+^CD38^+^ cells (right). Early apoptosis: Annexin V^+^7-AAD^−^; late apoptosis: Annexin V^+^7-AAD^+^. **I** Flow cytometry quantification of the HSC maturation markers CD133 and HLA class II in FL and UCB HSPCs. **J**–**K** RT‒PCR analysis of PROM1 (**J**) and ESAM (**K**) in FL and UCB HSPCs (n = 3), *p < 0.05, and **p < 0.01. **L** Flow cytometry analysis of CD144 in FL and UCB HSPCs

The first human HSCs are generated from arterial ECs via HSC-primed HECs through EHT in the AGM region [23, 24]. Afterwards, the first transplantable HSCs in the liver are detected at 7 weeks [43]. The FL is a major site for HSC amplification and differentiation. Most human FL HSPCs (CD34^+^CD144^+^CD45^+^ cells) lose the expression of endothelial-specific markers as they rapidly start expressing hematopoietic markers following HSC colonization to the liver [43]. Our proteome data demonstrated that highly validated fetal-biased HSPC proteins (such as LIN28B, IGF2BP2, and IGF2BP3) and endothelial-related proteins (such as ESAM [44–46] and ENG [47, 48]) were overexpressed in FL HSPCs (Fig. 7A/K). However, the overexpression of HSC maturation-related proteins (PROM1 [4, 24, 49], HEMGN [50], ALDH1A1, HOPX, and PTPRC) and HSC TFs (MSI2 and BCL11A) in UCB or aBM HSPCs indicated the maturational switch from the FL stage to the aBM stage (Fig. 7A). Early FL hematopoiesis exhibited erythroid lineage bias (HBE1 and HBZ, TRDC, and CD105), whereas most lymphoid and myeloid lineages were represented at later stages (Fig. 7A). This finding is in accordance with previous studies gained from scRNA-seq or functional assays [32, 51, 52]. Consistent with this, the colony numbers containing erythroid cells were significantly higher in FL Lin^−^CD34^+^CD38^−^ HSPCs than in neonatal and adult stages (Fig. 7B/C). The enhanced erythroid potential in FL is likely vital to establishing an effective oxygen transport system for the developing embryo. CFU assays also showed FL HSPCs exhibited more powerful in-vitro multi-lineage potential (CFU-GEMM) (Fig. 7B/C).

Beyond that, FL and UCB HSPCs exhibited increased ex vivo expansion potential than aBM HSPCs (Fig. 7D-F), which agreed with previous studies [53, 54]. Lin^−^CD34^+^CD38^−^ and Lin^−^CD34^+^CD38^+^ in G0 phage increased during hematopoiesis development, indicating the enhanced proliferative potential of FL HSPCs and a progressive shift to the quiescence state (Fig. 7G, Additional file 1: Fig. S7A). Meanwhile, the fractions of apoptotic cells in Lin^−^CD34^+^CD38^−^ and Lin^−^CD34^+^CD38^+^ populations in the FL stage were increased compared with their UCB and aBM counterparts (Fig. 7H, Additional file 1: Fig. S7B). The elevated level of ROS (Fig. 5L/M), decreased GSTs and reduced protection against ROS-induced protein oxidation (Fig. 4B-E) may be the reason for the increased apoptotic cells in human FL HSPCs. All these results demonstrated that human HSCs at different developmental stages show functional heterogeneity, including cell cycle state, multi-lineage and in-vitro expansion potential, which also verified the HSPC function characteristics and metabolic-related features derived from our proteomic data.

Proteins involved in antigen processing and presentation (such as HLA-A, HLA-B, and HLA-DRB4) were significantly upregulated in UCB and aBM HSPCs (Additional file 1: Fig. S7D). HSPCs constitutively present antigens via HLA class II, and compared with DR-negative cells, human HLA-DR^+^ BM cells give rise to significantly higher levels of multilineage engraftment after being transplanted into NSG mice [55]. We found an increase in the expression of HLA-DR/DP/DQ and CD133 in Lin^−^CD34^+^ HSPCs from FL to UCB, suggesting the involvement of signature surface markers during human HSPC developmental maturation (Fig. 7I). The significantly increased level of PROM1 (CD133) in UCB HSPCs was also validated by RT‒PCR (Fig. 7J). Moreover, the expression of CD144 decreased during HSPC development (Fig. 7L). UCB and aBM HSPCs were enriched in immune-related processes, such as the chemokine signalling pathway and T-cell receptor signalling pathway (Fig. 2D–E, Additional file 1: Fig. S3B/7C), which is consistent with the immune protection function of HSCs against pathogens after maturation. UCB and aBM HSPCs also expressed relatively high levels of proteins related to “DNA repair” and “DNA damage response” (Additional file 1: Fig. S3B/7E), which may prevent ROS-mediated oxidative stress [56, 57] (Additional file 1: Fig. S3B/7F). Altogether, the drastic and dynamic metabolic switch in HSPC maturation from the embryonic stage to the adult stage through the perinatal stage was illustrated.

## Discussion

Knowledge of HSCs and their metabolism has been obtained mainly from studies using mouse models and in vitro systems. During development, HSCs undergo a series of changes in number and function. HSCs also possess the ability to maintain their dynamic balance among quiescence, proliferation, and differentiation [58]. For example, embryonic HSCs have a more active cell cycle than adult HSCs [11]. ScRNA-seq revealed that genes related to HLAs were significantly upregulated during human HSC maturation [24]. Functional HSC activity is associated with HLA-II expression in human BM HSPCs [55]. Protein homeostasis is critical for stem cell maintenance, whereas HSC maintenance requires highly regulated protein synthesis [26, 59]. Cell metabolism consists of both anabolic and catabolic reactions, which support cell survival, fate decision, and function [5]. However, the metabolic-related pathways involved in HSPC maturation from the fetal stage to the adult stage remain elusive.

In this study, we performed proteomic analysis of human FL, UCB, and aBM HSPCs and revealed dynamic fetal-to-adult changes in HSPC development. Our study demonstrated that HSPCs adapt their metabolism to favour their different cell cycle state, ex vivo expansion and multi-lineage potential, and apoptosis during development. Our results indicated that FL and UCB HSPCs exhibited increased ex vivo expansion potential than aBM HSPCs. Previous studies showed that human BM and UCB CD34^+^ cells differ significantly in cell division kinetics and the expression CD34/CD38 during ex vivo expansion [60]. UCB CD34^+^ HSPCs showed higher proliferation and ex vivo expansion potential than BM-derived CD34^+^ HSPCs, possibly due to the ability of UCB HSPCs to exist more rapidly from G0-G1 phases [61] or the longer telomere length in fetal/neonatal HSPCs than adult HSPCs [62]. Human FL HSPCs presented increased OXPHOS levels and ribosome biogenesis to meet their increased metabolic energy requirements. In contrast, UCB and aBM HSPCs (especially in the aBM stage) preferred anaerobic glycolysis to maintain a relatively low metabolic status, quiescent state, and self-renewal potential. Human BM Lin^−^CD34^+^CD38^−^ HSPCs keep a low frequency of apoptosis under steady-state conditions, which could protect against damage accumulation [63]. Our proteomic data support the involvement of a metabolic switch from aerobic respiration to glycolysis upon HSPC maturation from the fetal stage to the adult stage.

In addition, UCB and aBM HSPCs presented lower protein synthesis levels for long-term hematopoiesis maintenance (Additional file 1: Fig. S5G). The decreases in mitochondrial activity, glucose uptake, and ROS production also pointed towards this metabolic switch. Compared with adult hematopoiesis, higher levels of cell metabolic activity, oxygen supply, and protein synthesis rates could meet the demand for cell division in the fetal stage. Our results demonstrated that increased metabolism levels in fetal HSPCs could promote high proliferation without disrupting their stemness ability. However, UCB and aBM (especially in the aBM stage) HSPCs display relatively lower OXPHOS levels, ROS production, and protein synthesis rates to preserve their self-renewal ability. We also provide proteomic and functional data indicative of an enhanced ability to counteract the effect of irreversible protein oxidation through increased expression of GSTs (such as GSTP1, LANCL1, GSTM2, and GSS) in human UCB or aBM HSPCs compared with their fetal counterparts (Fig. 4B–E). UCB and aBM HSPCs, which showed quiescent cell cycle and lower apoptosis, also expressed significantly greater levels of proteins involved in the cellular response to oxidative stress than did fetal HSPCs (Additional file 1: Fig. S3B/7F).

We also performed functional validation related to GSH metabolism. Lin^−^CD34^+^CD38^+^ HPCs exhibited far less apoptosis than immature Lin^−^CD34^+^CD38^−^ HSPCs did, indicating different responses to BSO-induced elevated ROS levels and reduced translation rates. Previous studies have demonstrated that murine fetal HSPCs are more susceptible to oxidative stress than adult HSPCs [64]. Our study demonstrated that human HSPCs are more vulnerable to BOS-induced oxidative damage than HPCs. Pan-antioxidant NAC treatment improved human HSC engraftment and multilineage hematopoietic differentiation in NOD/SCID mice [65]. Our results suggest that improving the ability to counteract oxidative stress may enhance HSC function and provide novel strategies for targeting specific antioxidative genes rather than applying pan-antioxidants. Our findings provide significant implications for designing strategies for the application of UCB and aBM HSPCs in the setting of hematologic malignancy transplantation, as well as the in vitro regeneration of human HSPCs.

Although scRNA-seq technologies have revolutionized our understanding of stem cell biology, little is known about posttranscriptional mechanisms and their associated networks. Our results demonstrated that the proteome is powerful for evaluating metabolic properties and changes. The metabolic heterogeneity of murine HSCs has recently been investigated at single-cell resolution [66]. Human HSCs are rare, CD34 is a marker of human HSCs and lineage-restricted progenitors [28], and the heterogeneity of these populations cannot be resolved via current proteome technologies. Because obtaining enough rare cell populations, such as human HSCs, is challenging, exploring their functions in subsequent HSC regeneration in vitro is limited. Our results indicated that metabolic pathway changes were primarily evident at the protein level, highlighting the power of proteomic analysis. Our data should be regarded as proteomic resources of the stem and progenitors of hematopoietic cells; the signatures indicate the commonalities shared with most of the subpopulations of human HSPCs. Notably, the mitochondrial activity of Lin^−^CD34^+^CD38^−^ (HSPCs) and Lin^−^CD34^+^CD38^+^ (HPCs) at different developmental stages exhibited a similar pattern (Fig. 5F–I), which was consistent with our proteomic analysis of the Lin^−^CD34^+^ HSPC population. Thus, proteomics methods must be optimized to accurately and precisely rare phenotypic cell populations, thereby highlighting the necessity of functional assays. Combining multi-omics analysis with functional data will lead to a better understanding of human HSPCs at different developmental stages.

The metabolomic landscape presented herein emphasized the molecular and metabolic differences between human FL, newborn, and adult Lin^−^CD34^+^ HSPCs. Compared with their UCB and aBM counterparts, highly proliferative FL HSPCs prioritize the usage of oxygen-dependent energy-generating pathways, as well as dynamic metabolism and ribosome biogenesis. The development and maturation of HSPCs, as well as the maintenance of homeostasis of HSPCs, are accompanied by metabolic remodelling, functional heterogeneity, dynamic changes in immune-related signalling, and antigen presentation. Disturbing glutathione metabolism resulted in distinct responses, including proliferation, apoptosis, and the cell cycle, between human Lin^−^CD34^+^CD38^−^ HSPCs and Lin^−^CD34^+^CD38^+^ HPCs. Further functional studies are required to assess the biological significance of these differences in the near future. In vitro-generated HSPCs cannot complete maturation, highlighting the importance of understanding the molecular program of in vivo HSC maturation and metabolic changes and developing advanced protocols to mimic emulation HSC niches. These discoveries and datasets represent valuable resources for further studies on hematopoietic system development and the mechanism contributing to haematological malignancies.

### Limitations of the study

Using high-depth proteomics analysis, this study characterized human FL, UCB, and aBM HSPCs. The experimental design and nature of human tissues and samples still come with several limitations, such as the fact that human embryonic tissue and blood samples are difficult to obtain. Despite the limited material, we performed several HSPC function validation (CFU, HSPC expansion, proliferation, and apoptosis), RT‒PCR assays, and metabolic-related assays to confirm the knowledge gained from our proteome data. In addition, cellularity varies significantly between individuals, which is common in human samples, both in physiological and pathological scenarios. The cell number required for proteome analysis of a sample can be excessive after taking data quality into account. We also believe in and look forward to the optimization of proteomics technologies to analyze rare cell populations with high accuracy and sensitivity, even at the single-cell level. Proteomic analysis of immunophenotypic HSCs and HPCs will be performed shortly. Our current data lay the foundation for further study.

## Supplementary Information

Additional file1

Additional file2

Additional file3

Additional file4

Additional file5

## Data Availability

The mass spectrometry proteomics data have been deposited in the ProteomeXchange Consortium (https://proteomecentral.proteomexchange.org) via the iProX partner repository with the dataset identifier PXD052196. The data supporting the findings of this study are available in the Additional files.

## Abbreviations

HSPCs: Hematopoietic stem progenitor cells
HSCs: Hematopoietic stem cells
FL: Fetal liver
UCB: Umbilical cord blood
aBM: Adult bone marrow
OXPHOS: Oxidative phosphorylation
ROS: Reactive oxygen species
AGM: Aorta-gonad-mesonephros
LT-HSCs: Long-term HSCs
HPCs: Hematopoietic progenitor cells
DEPs: Differentially expressed proteins
G-CSF: Granulocyte colony-stimulating factor
PBMCs: Peripheral blood mononuclear cells
LSCs: Leukemic stem cells
PCW: Post-conception weeks
CRL: Crown-rump length
MNCs: Mononuclear cells
MPPs: Multipotent progenitors
ECs: Endothelial cells
MTG: MitoTracker Green
DIA: Data-independent acquisition
PCA: Principal component analysis
BSA: Bovine serum albumin
PBS: Phosphate-buffered saline
KEGG: Kyoto Encyclopedia of Genes and Genomes
PPAR: Peroxisome proliferator-activated receptor
FCM: Fuzzy c-means
TCA: Tricarboxylic acid
PPP: Pentose phosphate pathway
AA: Amino acid
OCR: Oxygen consumption rate
PER: Proton efflux rate
HK1: Hexokinase 1
PYGL: Glycogen phosphorylases liver form
PGM2: Phosphoglucose mutase 2
ALDOC: Aldolase C
TPI1: Triosephosphate isomerase
ENO1: Enolase-1
ENO2: Enolase-2
RT-PCR: Reverse transcriptase-polymerase chain reaction
GLS: Glutaminase
GLUD1: Glutamate dehydrogenase 1
GSH: Glutathione
GST: S-transferases
EHT: Endothelial-to-hematopoietic transition
RA: Retinoic acid
2-NBDG: 2-[N-(7-nitrobenz-2-oxa-1,3-diazol-4-yl)amino]-2-deoxy-D-glucose
OP-puro: O-propargyl-puromycin
BSO: Buthinine sulfoximine
NAC: N-acetyl-L-cysteine
ESAM: Endothelial cell-selective adhesion molecule
ENG: Endoglin
HEMGN: Hemogen
HOPX: Homodomain-only protein
BMI1: Polycomb complex protein
MS: Mass spectrometry
Rot/AA: Rotenone/antimycin A
iRT: Index retention time
