# Supplementary material for: Proteomics reveals dynamic metabolic changes in human hematopoietic stem progenitor cells from fetal to adulthood
Source: Stem Cell Res Ther. 2024 Sep 15;15:303. doi: 10.1186/s13287-024-03930-x (PMC11403967; doi:10.1186/s13287-024-03930-x)

Supplemental Figure 1

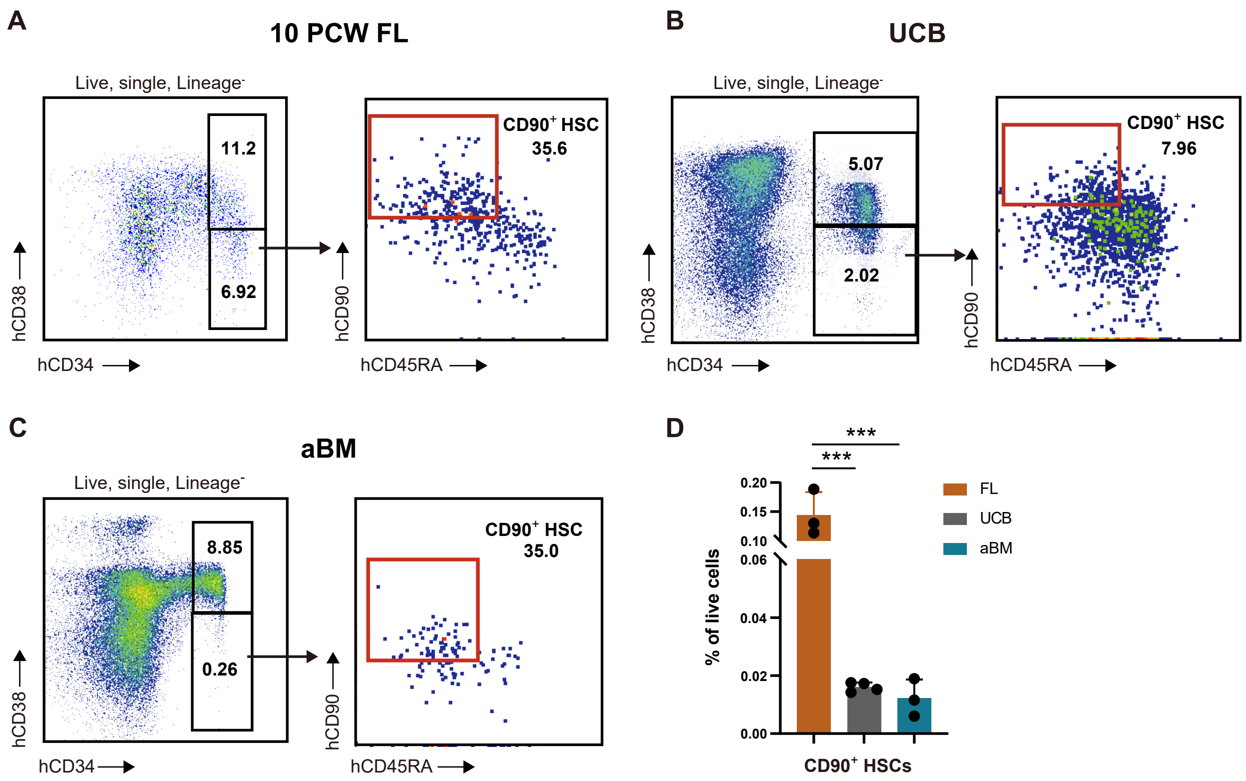

Supplemental Figure 2

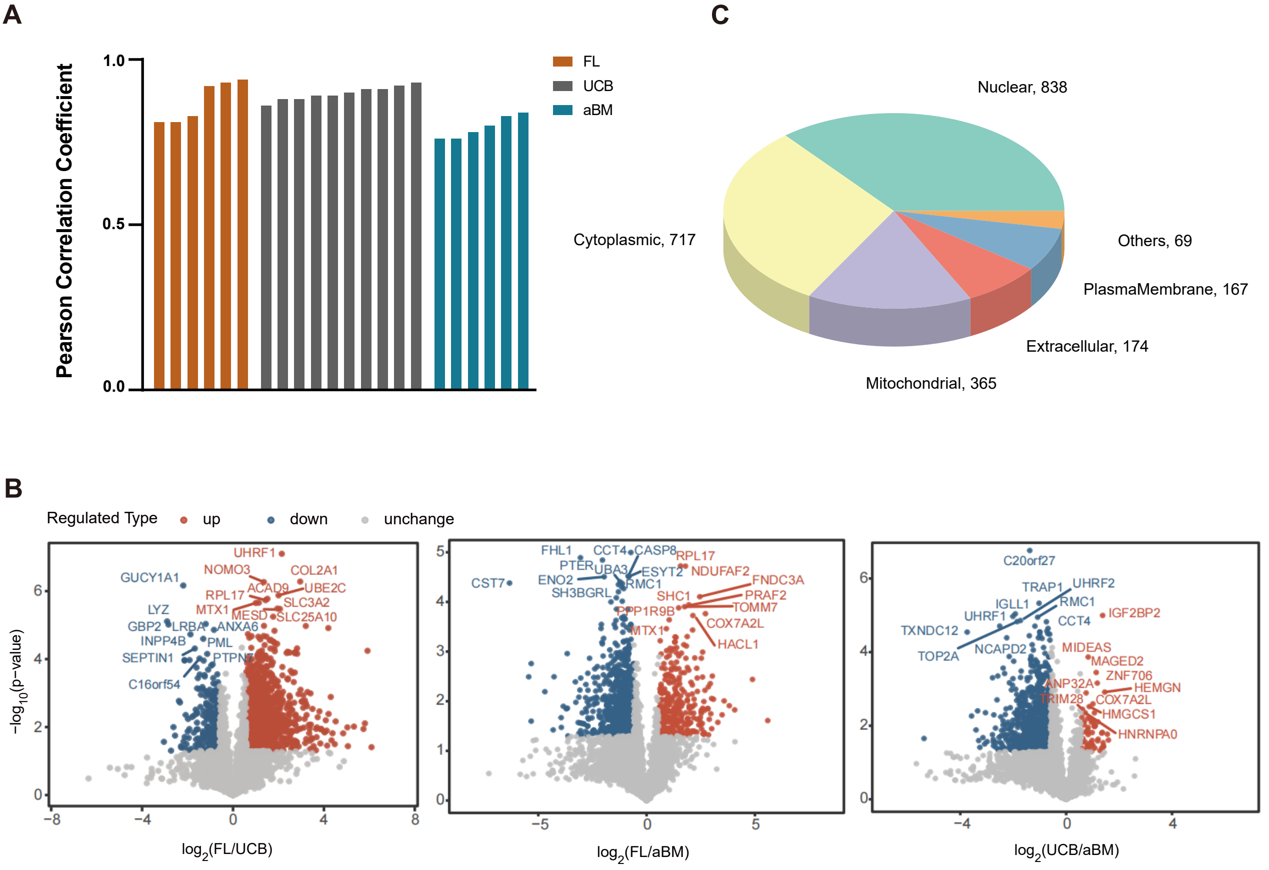

Supplemental Figure 3

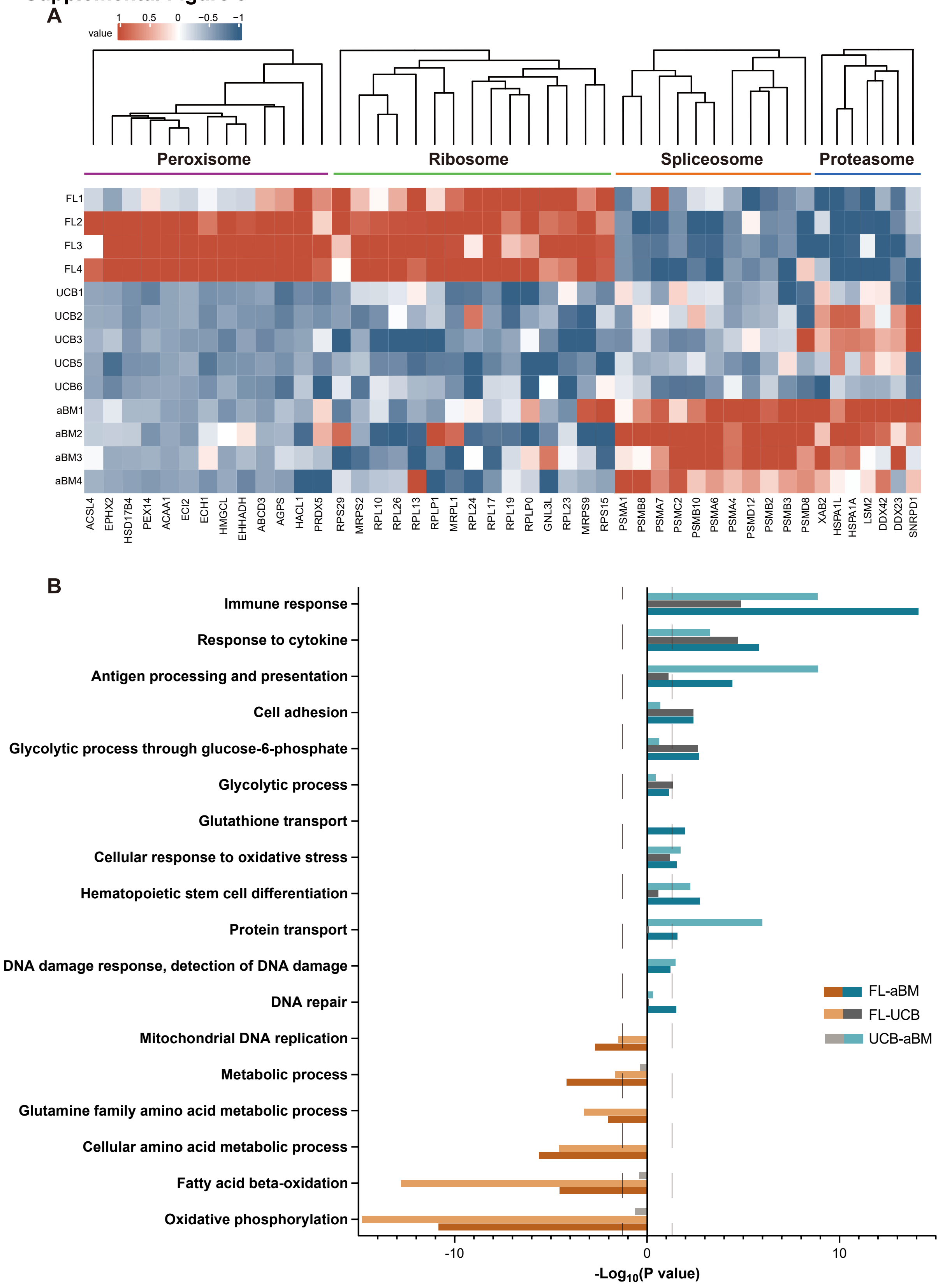

Supplemental Figure 4

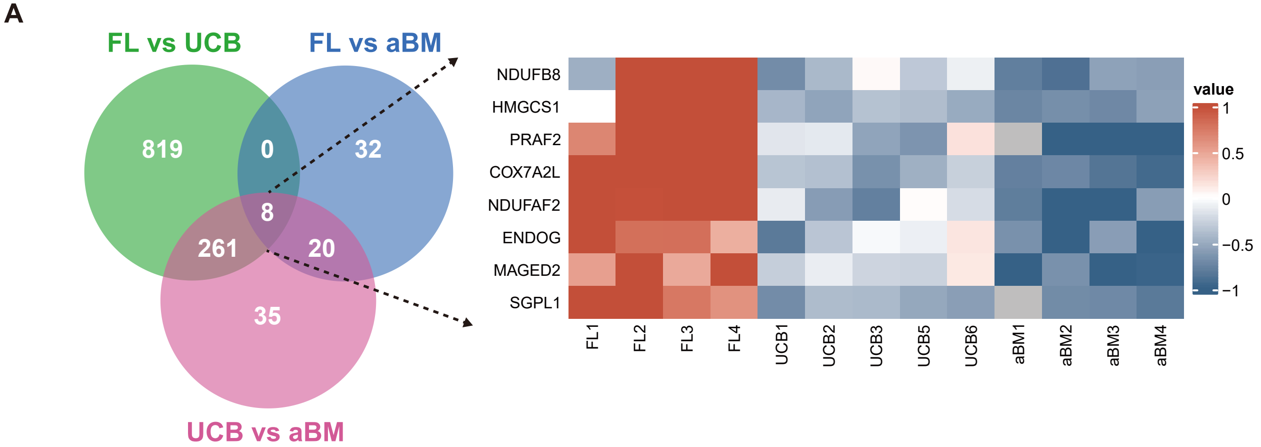

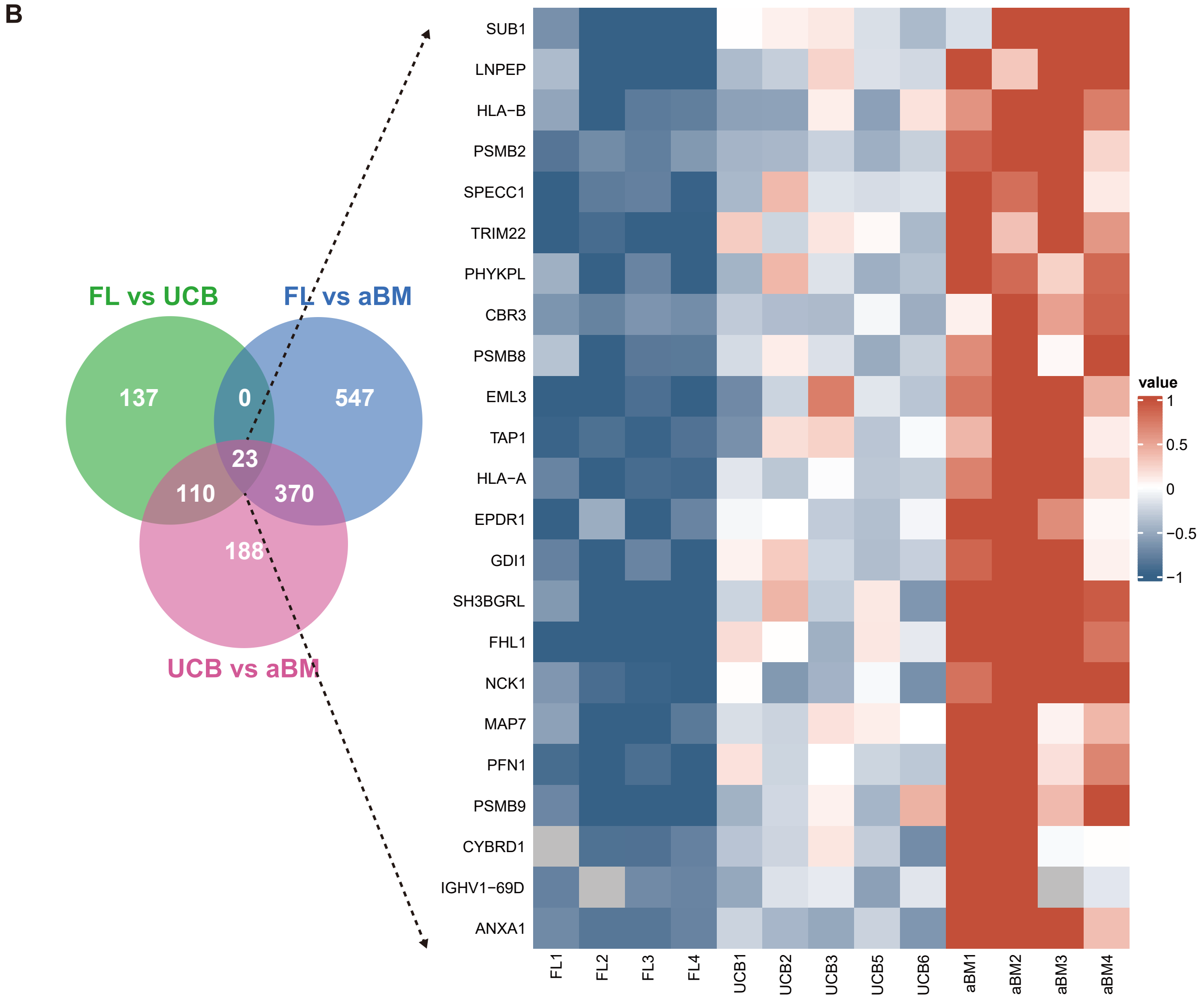

Supplemental Figure 5

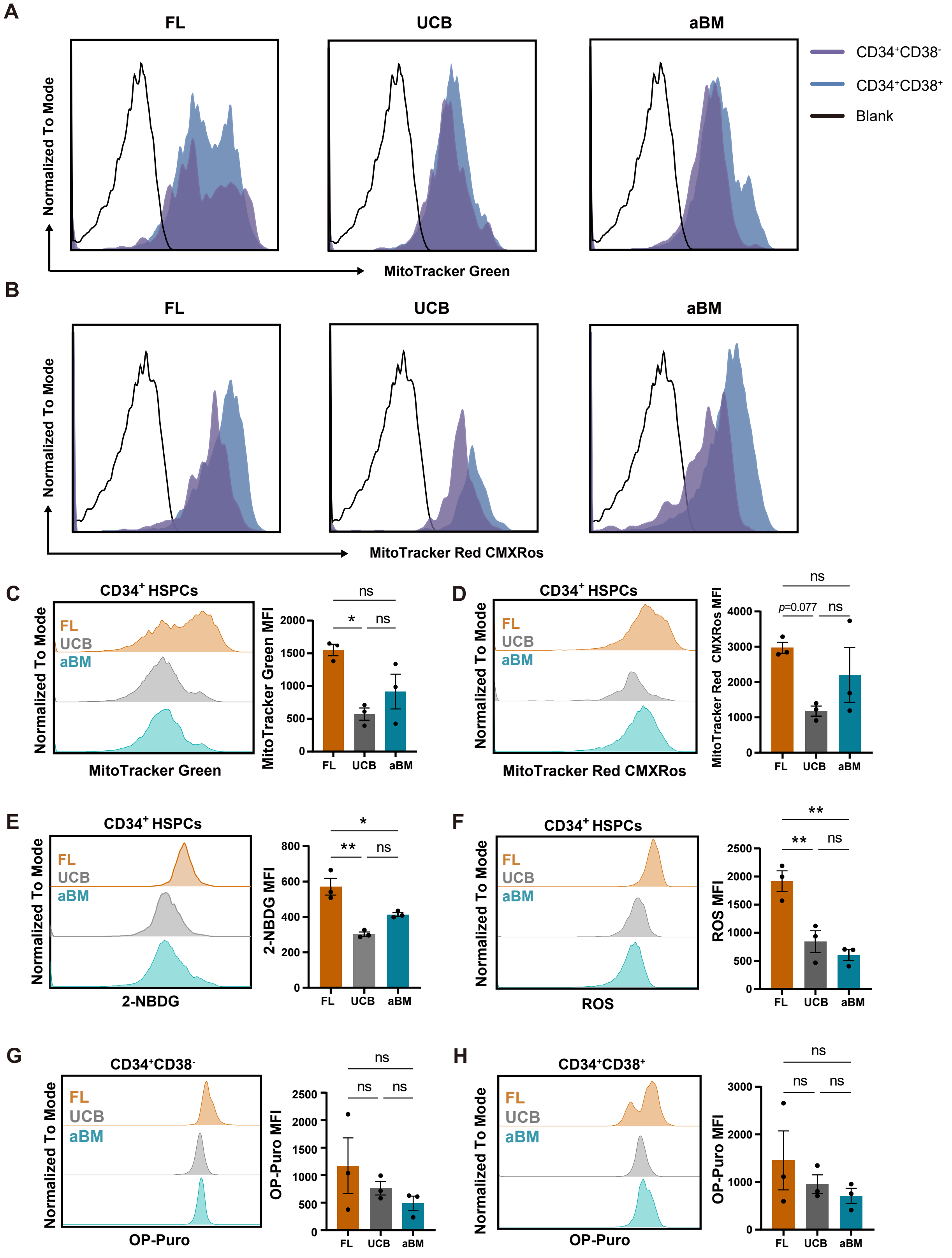

Supplemental Figure 6

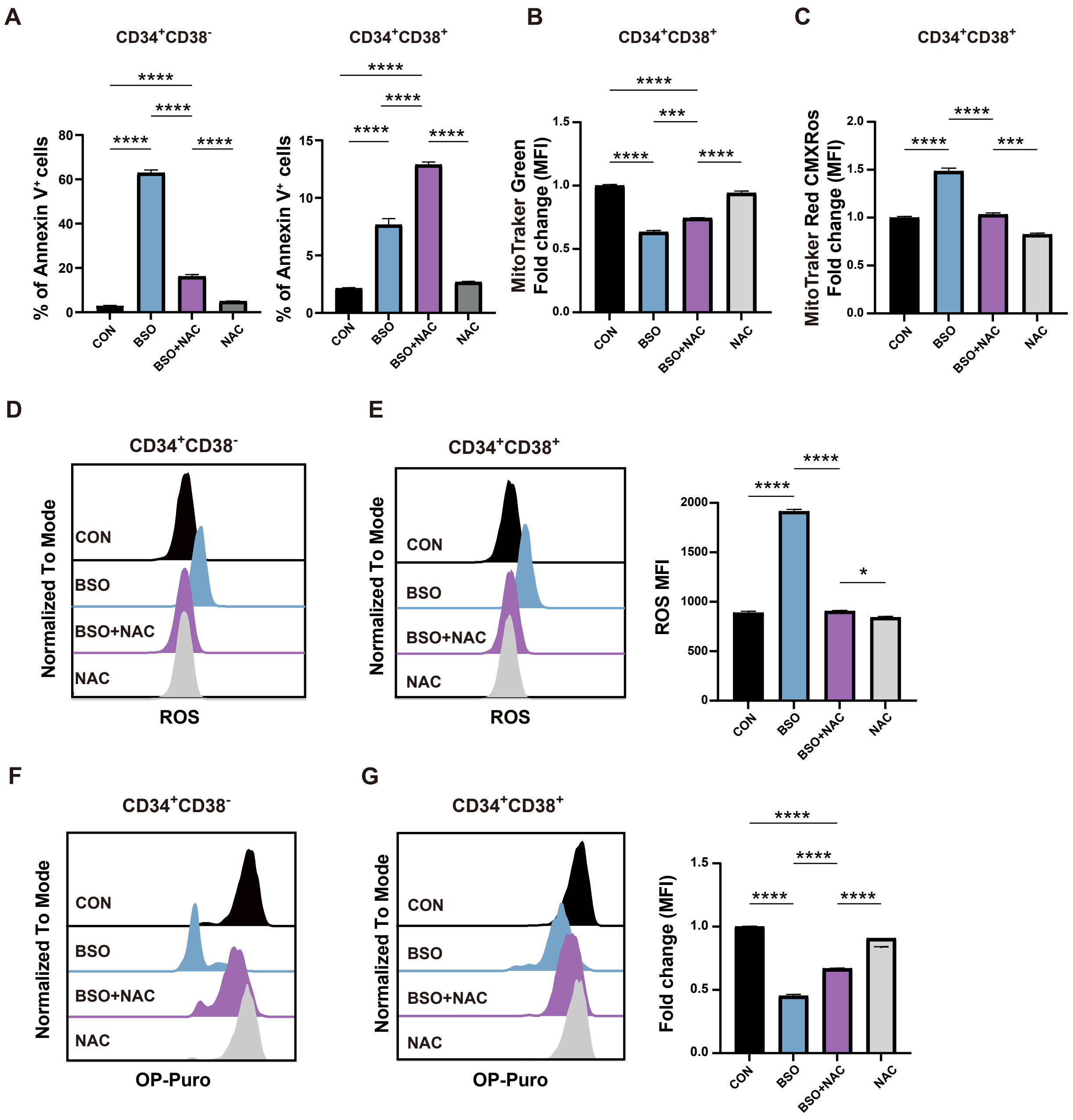

Supplemental Figure 7

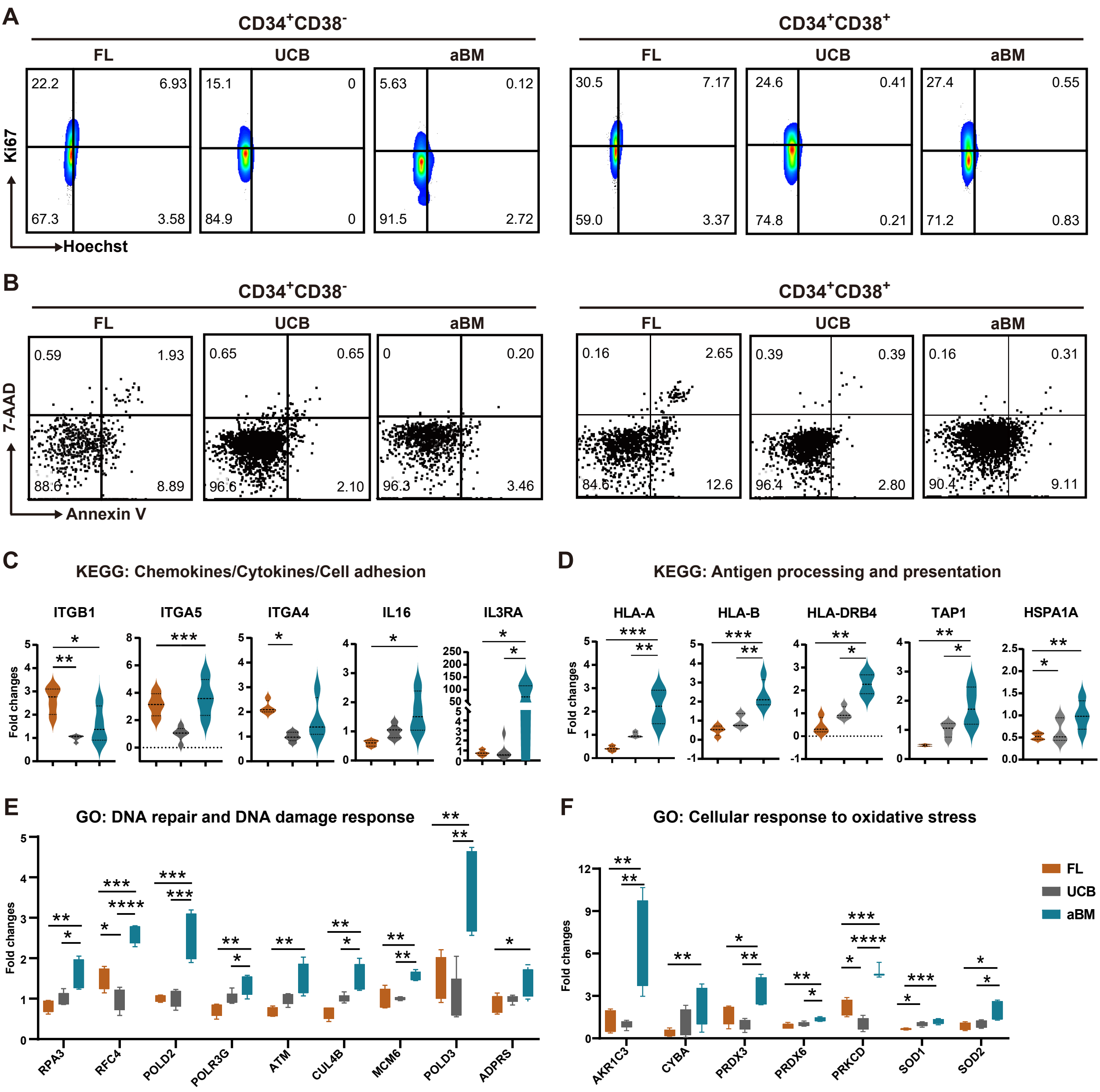

Supplement: Supplementary file 1 — Additional file1 [file 13287_2024_3930_MOESM1_ESM.pdf]
